# Overcrowding Indicators in Emergency Departments Across Countries: Scoping Review

**DOI:** 10.2196/78073

**Published:** 2026-05-05

**Authors:** Natasya Nur Mohd Nasir, Ku Anis Shazura Indera Putera, Nur Jihan Noris, Zalina Libasin, Muniamal Krishnan, Nor Fauziah Salaton, Kah Yee Lum, Nur Nadia Renu Abdullah, Intan Syafinaz Saimy

**Affiliations:** 1 Institute for Health Management, National Institutes of Health, Block B1, No.1, Jalan Setia Murni U13/52, Section U13, Shah Alam, Selangor, 40170, Malaysia, 60 33628338 ext 8338

**Keywords:** emergency service, emergency indicator, emergency department, overcrowding indicators, emergency department overcrowding, global emergency overcrowding, PRISMA

## Abstract

**Background:**

Emergency department (ED) overcrowding is a persistent global health issue associated with adverse patient outcomes, diminished staff performance, and compromised health-system efficiency. Despite widespread recognition of the problem, there is no universally accepted approach to monitoring ED overcrowding. The use of disparate, nonstandardized indicators hampers cross-country comparison and the development of effective policies. A comprehensive synthesis of indicators currently used is essential to guide the adoption of robust, evidence-based metrics across diverse health care settings.

**Objective:**

This study aims to identify, consolidate, and categorize indicators that have been used internationally to assess ED overcrowding and to highlight gaps in their use.

**Methods:**

A comprehensive scoping review was conducted from October to November 2023 using four databases: PubMed, Scopus, Emerald Insight, and Google Scholar. Studies were systematically searched using predefined eligibility criteria. Level 1 and 2 screening were independently conducted by 9 researchers (NNMN, KASIP, NFS, NJN, MK, ZL, NNRA, LKY, and ISS) to minimize bias and enhance reliability, and discrepancies were resolved by consensus. A third reviewer (ISS) performed a full-text review, synthesis, and descriptive analysis. Indicators were categorized into input, throughput, and output. Input refers to factors driving ED demand, throughput encompasses internal ED processes such as triage, diagnostics, and treatment, and output addresses challenges in transferring patients to inpatient beds, such as bed shortages or delays. Descriptive analyses were then used to consolidate these indicators and to establish their relative importance. They were ranked based on frequency of reporting across diverse countries and health care settings.

**Results:**

Out of 1347 articles screened, 117 articles were included in the study. A total of 307 indicators were retrieved and then consolidated into 26 distinct indicators. The majority of indicators were classified within the throughput domain (209/307, 68%), followed by the output domain (62/307, 20%) and the input domain (36/307, 12%). The most common throughput indicator, which was frequently reported, was ED length of stay, cited 87 times, followed by left without being seen and waiting time, each reported 30 times. Length of stay consistently emerged as a primary marker of systemic bottlenecks and operational inefficiencies across health care systems.

**Conclusions:**

This review indicates that throughput measures, particularly length of stay, dominate current approaches to assessing ED overcrowding, whereas input and output indicators remain comparatively underrepresented. By consolidating 26 distinct indicators from 117 studies, this study provides a comprehensive evidence base to support the standardization of metrics for monitoring ED overcrowding internationally. These findings offer practical guidance for policymakers and health care leaders seeking to refine performance indicators, enhance benchmarking, and evaluate interventions aimed at improving patient flow. Further research should prioritize validation of underused indicators and the development of composite measures that better capture the complexity of ED crowding across diverse health care settings.

## Introduction

### Background

Emergency department (ED) overcrowding is a significant challenge faced by health care systems globally [1]. Although various studies have proposed definitions, ED overcrowding fundamentally occurs when the department’s capacity is insufficient to meet the urgent needs of patients, leading to delays in care due to congestion [2]. Overcrowding is characterized by a surge in patients seeking care that exceeds the department’s capacity to provide timely and efficient services, which reflects a mismatch between patient demand and available resources [3]. Overcrowding in the ED can arise due to a variety of factors, including an increase in patient volume, staffing shortages, and inefficient workflows. The scenario of overcrowding often begins even before a patient sets foot in the ED, influenced by factors such as external medical systems, lack of timely access to primary care, or seasonal health surges [4].

It is also associated with a range of adverse outcomes, including longer treatment durations, preventable medical errors, and the proportion of patients who leave the ED without receiving a medical evaluation from a health care professional [5]. Addressing ED overcrowding effectively requires comprehensive monitoring of patient flow, guided by a well-structured system of performance indicators. Health care authorities rely on these quality indicators to assess ED performance during regular operations and identify opportunities for improvement. The selection of appropriate indicators is crucial, as they guide decision-making based on specific process factors and the outcomes they measure. Improper selection or implementation of these indicators can lead to unintended and detrimental effects [6].

Indicators are essential for addressing ED overcrowding by providing real-time, data-driven insights across the patient journey, from seeking care to discharge or admission, while also monitoring key quality metrics to ensure efficient care delivery [7]. Both operational and clinical indicators are vital for guiding decision-making at each level. Previsit indicators, such as emergency call volume trends, ambulance diversion rates, and regional health data, help predict demand surges. Once patients arrive, operational indicators like triage times, treatment delays, and bed availability ensure efficient resource allocation and timely care delivery. Monitoring these indicators is crucial for public health surveillance, as they provide a comprehensive understanding of overcrowding and inform evidence-based interventions [8]. By tracking specific indicators, health care authorities can identify emerging trends, proactively allocate resources, and implement strategies to prevent overcrowding, thereby improving patient outcomes and health care delivery. Similarly, an Australian ED is exploring the integration of patient flow indicators, including measures of patient acuity, length of stay, and the availability of beds, to improve patient care and manage overcrowding in the ED. The implementation of the “Emergency Department Throughout Time Indicator” in Australia has helped to streamline patient flow, reducing waiting times for critical care [9].

The need for appropriate ED overcrowding indicators is critical for enhancing the efficiency and effectiveness of emergency care systems [10]. By using these indicators, health care authorities can analyze key metrics, such as patient wait times, length of stay, and throughput rates, providing a more accurate understanding of patient flow dynamics and facilitating targeted interventions [11]. These indicators play a vital role in assessing resource use and availability, enhancing the understanding of overcrowding, and driving improvements in health care delivery, resource management, and patient outcomes in both global and local contexts [6].

### Study Objective and Rationale

The primary objective of this study is to describe the indicators used to measure overcrowding at the EDs in hospitals using a scoping review. This objective helps to review the indicator used across a range of hospital settings globally. To achieve this, the study is guided by the central research question: what indicators are used to measure overcrowding in EDs in hospitals worldwide? This question forms the basis of the investigation, which aims to provide a comprehensive understanding of how overcrowding is measured and managed across different health care environments. The findings are expected to contribute to the development of more effective strategies for addressing ED overcrowding and improving health care delivery on a global scale.

## Methods

### Study Design

This scoping review was conducted in accordance with the PRISMA-ScR (Preferred Reporting Items for Systematic Review and Meta-Analyses extension for Scoping Reviews) as presented in Checklist 1.

### Protocol and Registration

The study protocol was registered with the National Medical Research Registry, Malaysia, under NMRR ID-23‐01709-HDO. Data collection was carried out between May 2023 and April 2024. The content of this review adheres to the registered protocol and aligns with the stated study objectives. The authors are accountable for all aspects of the work in ensuring that questions related to the accuracy or integrity of any part of the work are appropriately investigated and resolved.

### Eligibility Criteria

The search strategy was developed using the population, intervention, and outcome framework. The study population comprised health institutions with operational EDs, where service congestion and overcrowding are frequently reported. The intervention was defined as the implementation, application, or evaluation of institutional or hospital performance measures. The outcome focused on the primary indicators used to assess ED overcrowding within these emergency care settings. The comprehensive set of keywords related to ED overcrowding and the indicators used to evaluate or manage it was used to identify relevant studies. All records retrieved from the initial search were screened and assessed for relevance according to predefined inclusion and exclusion criteria in the Textbox 1.

Textbox 1. **Eligibility criteria.**

**Inclusion Criteria**

Studies were included if they met the following criteria:

- **Population:** conducted in hospitals, health centers, health institutions, emergency departments (EDs), trauma centers, or accident and EDs.
- **Intervention:** investigated indicators, measures, indices, parameters, attributes, determinants, or metric indices related to ED performance.
- **Outcome:** reported outcomes associated with overcrowding, congestion, or service overload within ED settings.
- **Study design:** primary research studies of any design, including reports addressing ED overcrowding.
- **Publication type:** article published in peer-reviewed journals.
- **Language:** published in English.
- **Access:** full-text articles available for review.

**Exclusion Criteria**

Studies were excluded if they met any of the following criteria:

- **Population:** not focused on EDs or involved nonhuman populations or nonhealth care facilities (eg, veterinary hospitals).
- **Intervention:** did not include any indicators or measurements related to ED overcrowding.
- **Outcome:** did not address issues of overcrowding, congestion, or overload.
- **Study design:** nonprimary studies such as literature reviews, commentaries, study protocols, or ongoing research.
- **Publication type:** other publication types, including conference papers, dissertations, or abstract books.
- **Language:** published in languages other than English.
- **Access:** full-text article not available.

### Information Sources

Articles were sourced from selected databases, including PubMed, Emerald Insight, Google Scholar, and Scopus. Google Scholar was included to capture gray literature, as the initial search yielded a substantial number of documents, particularly theses and technical reports. All retrieved records were assessed for eligibility.

### Search Strategy

The search process was conducted by the principal investigator, KASIP, in June 2023. This process targeted studies published between 2013 and 2023 to ensure the inclusion of the most recent evidence, considering health care system advancements [12]. The inclusion timeframe was restricted to the past 10 years to capture the most recent evidence, considering the rapidly evolving health care landscape that may limit the applicability of older studies [13]. A research specialist reviewed and optimized the search strategy, focusing on Boolean operators and database-specific indexing terms. Keywords were combined into final search strings, such as (hospital OR health OR healthcare) AND (indicators OR measure OR index) AND (overcrowding OR congestion), which were applied across all databases. The number of records retrieved and the corresponding search strategies are summarized in Table S1 in Multimedia Appendix 1. All results were exported to Mendeley, and duplicates were removed manually. The final set of articles meeting the inclusion criteria was incorporated into the review.

### Study Selection

Selected studies that capture literature on ED overcrowding and relevant indicators based on the eligibility criteria Textbox 1 were managed using Mendeley software by KASIP and LKY. The studies were evenly distributed among 4 pairs of researchers (Group 1: NJN and NNRA, Group 2: NNMN and ISS, Group 3: ZL and LKY, and Group 4: KASIP, NFS, and MK). Nine researchers (NNMN, KASIP, NFS, NJN, MK, ZL, NNRA, LKY, and ISS), working in pairs, independently reviewed the same articles to ensure accuracy and consistency. In most cases, each pair included at least 1 researcher with a clinical or public health background. Specifically, KASIP is a research officer, NNRA is a nurse researcher, and LKY is a pharmacist. During the level-1 screening process, researchers reviewed the titles and abstracts of the studies, and the required information was recorded using the Google Form. In level-2 screening, the process involved a detailed evaluation of the full texts of the studies that passed the first stage, with results recorded in a Google Sheet. Disagreements between paired reviewers at both stages were initially resolved through discussion and consensus. If the consensus was not achieved, a third independent reviewer (ISS) from a different screening pair, who had not previously screened the study, acted as the assessor to make the final decision.

### Data Charting

During level-1 screening, data collected primarily included study characteristics such as focus on ED overcrowding, document type, study design, publication status, and the presence of reported indicators. Upon completion of this stage, findings were mapped based on the presence of indicators or other measurements, and studies reporting relevant data were selected for level-2 screening. Level-2 screening focused on more detailed information, including year of publication, authorship, study setting, study design, study objectives, study acceptance, identified indicators, and the specific measurement tools used by 4 pairs. Findings from both screening levels were cross-checked, and any discrepancies were resolved through consensus discussions between reviewers to ensure rigor, validity, and trustworthiness. KASIP made the final determination, which was subsequently validated by ISS.

### Data Items

A list of data items was developed by KASIP and subsequently validated by ISS. These items are presented in Table 1.

**Table 1.** Data items extracted at each screening level.

Level of screening	Data items
Level 1	Focus on ED^a^ overcrowding (yes, no, or unable to retrieve the abstract) Document type (eg, articles in a journal: review, letters, editorial or comments, consensus statement, newsletter, original article, policy brief, thesis, or report) Study design (eg, case control, case study, cohort, cross-sectional, mixed methods, qualitative study, quasi-experiment study, randomized controlled trial, reviews – scoping or systematic and literature) Publication status - ongoing studies or protocols (yes or no) The presence of reported indicator (yes or not applicable)
Level 2	Article ID (code assigned to each selected study) Year of publication List of authors Study setting (origin or site of the study implementation) Study design Study objectives Acceptance of the study (accept or reject) Identified indicators Specific measurement tools (eg, National ED Overcrowding Study (NEDOCS) and Emergency Department Work Index (EDWIN)

a ED: emergency department.

### Critical Appraisal

Critical appraisal was not conducted and no risk of bias assessment of the included studies was undertaken in this study, as the purpose of a scoping review is to comprehensively gather and map existing evidence from the literature, summarize key concepts, and identify available findings across different contexts and countries that can inform and guide future strategies.

### Data Synthesis

Data extracted from both screening levels were categorized into input, throughput, and output components by KASIP, LKY, MK, and NJN, based on Asplin et al [14]. Input refers to factors that drive demand for ED services, including emergency, unscheduled, and safety-net care. Throughput encompasses ED activities such as triage, diagnostic evaluation, treatment, and inpatient boarding, while output addresses challenges in transferring admitted patients to inpatient beds, including limited follow-up care, bed shortages, or transfer delays. Using this concept, specific indicators of ED overcrowding, such as waiting time, length of stay, bed occupancy rate, and patient flow, were systematically extracted and organized to identify the most frequently used measures globally. This approach helps identify the bottlenecks, allowing the appropriate indicators and the planning of targeted strategies to improve the affected areas.

## Results

### Study Selection

**Figure 1.**
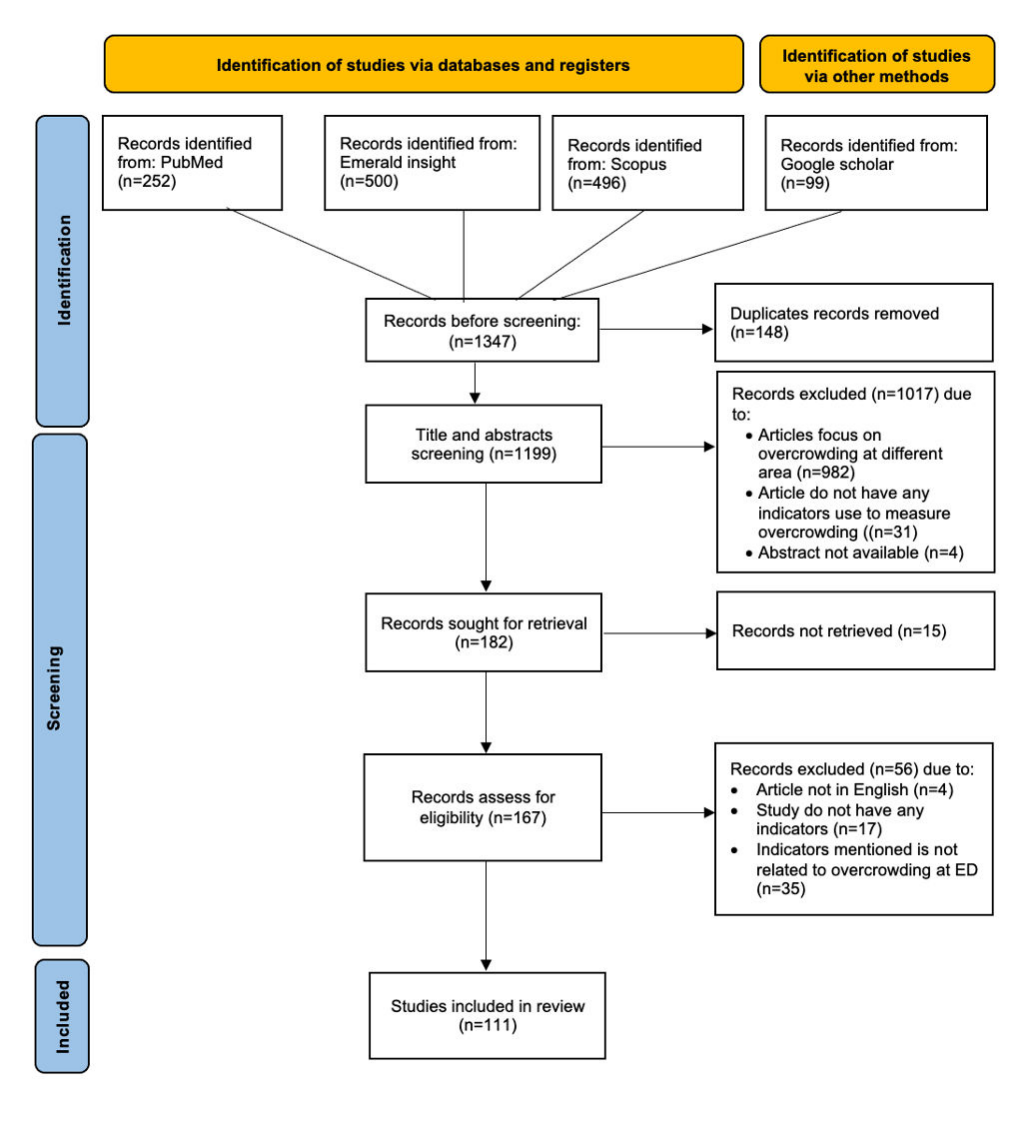
Flowchart of the article selection. ED: emergency department.

Figure 1 illustrates the process of identifying, screening, and inclusion of studies for the review. Initially, a total of 1347 records were identified from 4 databases: PubMed (n=252), Emerald Insight (n=500), Scopus (n=496), and Google Scholar (n=99). After removal of 148 duplicate records, 1199 titles and abstracts were screened. Of these, 1017 were excluded for the following reasons: focus on overcrowding in other areas (n=982), absence of indicators measuring overcrowding (n=31), or unavailability of abstract (n=4). Subsequently, 182 records were sought for retrieval, but 15 were unable to be retrieved. Next, 167 records were assessed for eligibility based on inclusion and exclusion criteria, of which 56 were excluded as the article was not in English (n=4), study did not report any indicators (n=17), or the indicators were not related to ED overcrowding (n=35). In the final level, 111 studies [3,7,11,15-121] as mentioned in the List of indicators in Multimedia Appendix 2, were included for data synthesis.

### Characteristics of the Included Studies

The greatest number of studies examining indicators was published in 2014 (n=14) [15-27, 109] followed by 2019 and 2021 (n=14 each) [1,3,28-51,122], 2017 (n=13) [52-63,123], 2016 and 2018 (n= 9 each) [7,64-80], 2020 (n=11) [81-91], 2022 (n=9) [92-100], and 2013 (n=6) and 2015 (n=6) each) [11,101,103-108,110-113]. Studies mostly originated from the United States of America (n=32) [16-18,22,24,28,34,39,40,44,51-54,68,72,77,80,82,83,87,89,96,103,104,107,109,112,114-117] [27]. Additional articles included in this study came from countries such as Australia (n=12) [23,26,31,36,47,55,56,73,75,76,98,122], Canada (n=7) [20,32,45,57,58,78,85], Italy (n=6) [29,37,43,59,92,99], The Netherlands (n=6) [7,33,79,101,111,117], Taiwan (n=6) [3,60,69,88,94,106,118], Sweden (n=4) [35,38,49,100], South Korea (n=5) [25,81,86,105,113], France (n=4) [11,30,71,95], India (n=2) [1,41], Israel (n=3) [48,95,108], Turkey (n=2) [15,19], Brazil (n=2) [42,52], England (n=1) [70], Germany (n=2) [21,93], Saudi Arabia (n=2) [46,90], Switzerland (n=2) [119,120], Belgium (n=1) [123], Iran (n=1) [61], and Vietnam (n=1) [50]. In some studies, the country was not specifically mentioned or not applicable (n=9) [62-65,67,74,91,102,106].

The study designs of the articles primarily included quality improvement, case-control, case study, cohort study, simulation modeling, cross-sectional study, quantitative study, editorial, literature or systematic or scoping review, mixed methods, retrospective study, quasi-experimental study, and time series. For the study design, most studies used a cross-sectional study design (n=55) [1,15-17,19,21-26,29,30,33,35,38,41-44,49-52,55,57,58,61,63,66,70,73,77,78,81-83,85,86,90,93,94,98,100,101,103-105,110-114,118,123], followed by a cohort (n=18) [7,11,20,28,31,32,45,46,48,53,54,56,60,69,79,89,112,116]. Detailed characteristics of the studies included in this review are shown in Multimedia Appendix 2.

### Result of Indicator Retrieval

Overall, 111 studies [3,7,11,15-121] reported indicators for measuring overcrowding, either alone or in combination with other tools, while 6 studies used only specific tools without reporting indicators. The list of indicators mentioned in Multimedia Appendix 2, a total of 360 indicators were initially extracted. The minimum number of indicators reported in the studies was one, whereas the maximum number of indicators identified in a single study was 11. Indicators that conveyed similar information and descriptions were identified and merged. Following detailed discussions among the investigators, indicators that conveyed similar information or measured the same underlying concept were grouped together to minimize redundancy. This results in a reduced set of 307 indicators.

These indicators were subsequently analyzed and categorized according to Asplin et al [14] conceptual model of ED overcrowding. Overall, out of 307 indicators identified, the majority, 209 (68%), were classified within the throughput component, reflecting their central role in managing ED performance. In comparison, 62 (20%) indicators were categorized in the output component, while the least proportion, 36 (12%) indicators, were categorized as input. Among the 307 indicators, several of them convey and measured the same parameters. They were then consolidated once more in the final phase of the analysis and presented as a single description for clarity and easier stakeholder comprehension. This process produced 26 distinct indicators.

### Synthesis of Results

The findings indicate that the widely used indicator to measure overcrowding was the strengthening of throughput processes, with ED length of stay (ED LOS) emerging as the most frequently used indicator. This was followed by waiting time and left without being seen (LWBS), each reported in 30 studies [19,20,22-24,28,30,34,43,54,57,65-67,70,73,85,102,104,110,114,115]. Within the input component, the average number of patients visiting the ED was the most commonly applied indicator. For output, ED boarding time was most frequently used. The results from the search strategy for indicators using PubMed, Emerald Insight, Google Scholar, and Scopus, and the frequency of indicator uses across studies, along with their classification according to ED components, are summarized in Table 2.

**Table 2.** List of indicators with frequency, and ED^a^ components classification.

Indicators	Description	Frequency mentioned (n)	Study	ED components
Average number of patients visiting ED	Average total number of patients who attended ED [91,114]	23	[7,15,16,27-29,41-43,57,62,64,73,81,101-103,114]	Input
Representations to ED within 48‐72 hours	Percentage of patients making an unplanned visit to ED within 48‐72 hours after first visit for the same chief complaint [119]	7	[15,65,66,104]	Input
Ambulance diversion or turnover time	Period during which ED-requests ambulances that would normally bring patients to the hospital to instead proceed to other hospitals presumed to be less crowded [52,105]	6	[17,30,31,55,102,106]	Input
ED length of stay	Measured from first clinician contact in the ED (triage or ED reception) until the patient physically left the ED (admitted, transferred, or discharged) [2,11,44,61]	84	[3,7,11,15,17-21,24,25,27,30,32-38,41-43,45-48,52-60,63-79,82-87,92-97,101,102,104,107-111,114-119]	Throughput
Waiting time	Total time from initial registration or triage to first being seen by a doctor or clinician [20,111]	31	[7,11,18,28,39,43,44,47,49,50,55,58,65,66,70,73,75,85,98,103,106,114,120] [100]	Throughput
Left without being seen	The number of patients who arrived, registered, and underwent the triage process but leave prior to being seen by a medical provider or physician [2,17,74,92]	30	[19,20,22-24,28,30,34,43,54,57,65-67,70,73,85,102,104,110,114,115]	Throughput
ED occupancy rate	The number of occupied beds divided by the total number of ED beds, expressed in percentage [15,24,88,92,112]	21	[25,26,31,35,38,41,42,44,46,61,73,80,89,90,92,96,99,102,105,112,113] [100]	Throughput
Arrival to consultation	Time taken by patient from arrival at primary triage until patient sees a doctor or first consultation by physician [30,65]	11	[11,30,58,72,83,85,87,97,103,107,111]	Throughput
Time to admit and treat	Time interval spent by patient between arrival in the ED and his or her first consultation by a physician [23,49,80,89,92]	7	[16,18,29,39,44,51,55]	Throughput
Time to decision and referral	Time measured from patient arrival to decision of referral, discharge, or admission [20,23]	5	[11,48,55,60,121]	Throughput
Ratio of doctors/patient, nurse/patient	Number of patients each unique clinician is responsible for in a given time slot, presented for each profession [39]	5	[38,92]	Throughput
Mortality rate	Refers to a patient’s death when that patient was still physically in ED or had been initially admitted to the hospital from ED, expressed in number [74]	4	[35,66,91,104]	Throughput
Patient satisfaction	Total percentage of patients ranked their overall ED care as very good or excellent [17,34]	4	[17,34]	Throughput
Diagnostic turnaround time and time after diagnostic test	Durations of the different diagnostics that a patient is subjected to during their stay in the ED. Defined as the time from order entry to the time the radiology and laboratory releases a report in the system [51]	3	[33,115]	Throughput
Percentage of patients left against medical advice	Patients who left without completion of medical care [15,111]	2	[41,83]	Throughput
Time to triage	Measured from patient’s arrival to first encounter with triage nurse [11,23]	2	[11,48]	Throughput
Number of ED beds	Number of licensed ED beds, as presented to state health office [92,94]	1	[16]	Throughput
Patient throughput	Number of patients who are treated and discharged from a health care facility within a specific period [93]	1	[18]	Throughput
ED boarding time	Time duration between a patient’s bed-request and admission to an inpatient bed [31,33,45,73,97,105]	25	[7,11,16-18,26,29,40,41,43,44,55-57,62,63,70,73,81,88,96,98,102,114,115]	Output
Number of admissions	Number of admissions from ED, represent workload level in ED [121].	18	[16,22,26,29,42-44,51,52,73,88,92,98,114]	Output
Discharge time	Time taken from decision to discharge by physician until patient leaves the bed [30]	9	[11,26,28,50,51,66,97]	Output
Percentage of patients exiting the ED within 4‐8 hours	Proportion of all patients exiting ED within 4‐8 hours, either discharged directly from ED or admitted to inpatient wards [5,119]	7	[11,18,23,56,62,91,111]	Output
Hospital Bed Occupancy Rate (BOR)	Ratio of occupied beds to census beds ratio [85,89] The number of patients in licensed bed and overflow locations over the number of licensed treatment beds [26]	5	[18,31,52,62,102]	Output
Bed turnover time	Time from when a patient is discharged until an inpatient bed is available for the next patient [3]	2	[52]	Output
Bed waiting time	Time from decision by ED doctor for admission and referral to primary team until patient arrives at bed in ward [30]	2	[7,97]	Output
Number of hospital beds	Number of licensed hospital beds, as presented to state health office [92,94]	1	[16]	Output

a ED: emergency department.

These 26 indicators' descriptions were collated and summarized as seen in Table 2. Categorization into the 3 components of ED crowding showed that the largest proportion of these indicators was concentrated within the throughput component, comprising 15 indicators, followed by the output component with 8 indicators, while the input component accounted for only 3 indicators. These results highlight the predominant representation of throughput elements compared to input and output elements, underscoring their central importance within the framework.

## Discussion

### Principal Results and Comparison With Prior Work

In this review, the numerous indicators used to assess overcrowding in the EDs illustrated the complexities and diversity of the ED system around the world. The methods by which these indicators are categorized also revealed differences in the conceptualization of overcrowding in the literature. Based on the framework by Asplin et al [14], the indicators obtained from the review were mapped to the input, throughput, and output components. Most of the indicators obtained in this review measured the throughput component, highlighting the importance of examining internal ED work processes, including triaging, room placement, initial provider evaluation, diagnostic testing, and treatment [7].

Throughput indicators dominate the ED performance system largely due to regulatory mandates, policy initiatives, and their alignment with multiple stakeholder interests. The Centers for Medicare and Medicaid Services has specifically made their metrics related to throughput measures for compliance and reimbursement purposes [124]. Furthermore, these metrics are relatively easy to capture from administrative data and electronic health records without the need for resource-intensive data collection methods [1,125]. Hospital administrators find the throughput metrics relevant due to their interest in operational efficiency and capacity management. Patients and families experience visible evidence of emergency performance through waiting times and length of stay, which made throughput measures the dominant focus compared to other quality dimensions [2,125].

Throughput components are mainly involved in internal processes in the ED. The overcrowding causes are due to the need of the specialists or consultants, a lack of instrumental diagnostic support, and a high number of procedures conducted, which results in a burden in ED and compromises the patient flow [4]. The strength of the manpower and adding resources could help in overcoming the overcrowding issues and fulfill the demand. The strategies from micro-level and macro-level should be managed appropriately based on the local setting and the health care institution’s capability [3,4,126].

Among the 3 components, ED LOS from the throughput category emerged as the most commonly measured indicator across studies. Introduced as part of the National Health Service ED performance metrics in the United Kingdom, length of stay serves as a critical measure of throughput and overcrowding, reflecting delays in diagnostics and treatment. Research has shown that patients discharged during shifts with a mean Length of Stay ≥6 h face a 79% higher risk of 7-day mortality compared to those with a length of stay <1 hour [117]. Prolonged length of stay underscores systemic issues, such as diagnostic delays and bed shortages, highlighting its impact on patient outcomes. Reducing length of stay is vital for improving patient satisfaction and increasing the ED’s capacity to manage higher patient volumes. Prolonged length of stay is associated with higher patient dissatisfaction with ED care. This finding is supported by a study by Parker and Marco [127], which explored the correlation between length of stay and patient satisfaction. The authors discovered that patients were dissatisfied with ED services when the Length of Stay exceeded 195 minutes, whereas they were satisfied when the length of stay was less than 150 minutes.

The throughput indicators, often associated with regulatory and accreditation requirements, are integral to ensuring compliance and improving health care delivery standards [7]. Among these, waiting and boarding times have been recommended as routine quality measures. Waiting time, in particular, is a critical indicator with significant implications for patient satisfaction and perceived quality of care. A prospective cross-sectional study involving 644 patients found that increased waiting room time was associated with perceptions of compromised care (OR [odds ratio] 1.05, 95% CI 1.02‐1.09 for each additional 10 minutes spent in the waiting room), as well as receiving care in hallways (OR 2.02, 95% CI 1.12‐3.68) [41]. These perceptions can affect patient trust and the overall reputation of health care facilities [93]. Furthermore, prolonged waiting times can lead to dissatisfaction and health deterioration, potentially resulting in more severe complications. As a visible aspect of overcrowding, waiting time plays a pivotal role in shaping public perception and trust in health care services.

LWBS and ED occupancy rates are good proxies of patient satisfaction [11,18]. The LWBS rate reflects patient frustration and the ED’s inability to manage patient flow effectively. Studies have consistently documented a positive correlation between ED overcrowding and LWBS rates [11,32,38,93,96]. The number of patients leaving without receiving care varied widely, ranging from 213 over an 18-day period [18] to 14,170 over 27 months [28]. This trend highlights systemic inefficiencies within EDs that compromise patient safety and satisfaction [16]. High LWBS rates indicate long wait times and overcrowded conditions, posing significant challenges to operational efficiency, patient safety, and care quality.

Given the impact of patient satisfaction, EDs are increasingly focusing on improving internal processes and addressing overcrowding. This shift has driven the measurement of additional throughput indicators to inform strategies for enhancing patient flow and overall service quality [19]. This explains why these indicators are frequently measured for overcrowding in this review. In contrast, only 12% (36/307) of the indicators fall under the input component, with the average number of patients visiting the ED being the most common. Likewise, 20% (62/307) of indicators were categorized as output like ED boarding time and number of admissions. Since input indicators are related to the demand for ED services, and output indicators are influenced by the inefficient disposition of ED patients, these processes are more challenging to control. Some studies have identified patient arrival characteristics as influences of input indicators, while the inability to transfer patients out of ED affects output indicators [65,92]. These factors are outside the control of the ED and involve other departments, thus requiring greater coordination.

While throughput measures are important, they must be balanced against input and output indicators to avoid a narrow assessment on the performance of ED overcrowding. Since throughput measures primarily emphasize speed, they risk incentivizing premature discharges to meet time targets. In systems dominated by throughput metrics, other critical dimensions such as clinical quality and patient safety indicators may be overlooked [5,128,129].

### Limitations

This review has several limitations. First, the literature search was conducted in June 2023; additional studies may have been missed. Despite broad eligibility criteria, some relevant studies (eg, non-English or without full-text) may have been excluded. Nevertheless, the compilation and categorization of indicators remain relevant. Second, a key challenge was the lack of clear definitions for certain indicators, such as distinguishing between waiting time and the interval from arrival to consultation. However, the researchers have clearly defined the variables in the results to assist the reader in better understanding. Third, the heterogeneity of indicators across studies also made direct comparisons difficult. Fourth, as no single “perfect” indicator exists, hospital systems may benefit more from using a combination of measures, such as ED occupancy rate, length of stay, and ED volume, to obtain a comprehensive assessment. Fifth, the variability in indicator use across settings complicates efforts to establish universal benchmarks. Sixth, intergroup reliability was not assessed, as the primary objective in this study was to identify indicators and map the findings according to input, throughput, and output components. Methodological rigor was maintained through independent screening, structured consensus discussions, and resolution by a third reviewer. These limitations highlight the need for more standardized definitions and consensus on evidence-based indicators to support meaningful comparisons and guide public health policies.

### Conclusions

In conclusion, the findings of this review highlight that throughput indicators were more prominently studied as key metrics in measuring ED overcrowding. The review provides further evidence that length of stay emerged as a critical indicator reflecting systemic bottlenecks and operational inefficiencies.

These findings underscore the importance of prioritizing throughput indicators, particularly ED LOS, as a benchmark for monitoring and addressing overcrowding. However, the lack of standardized definitions and variability in indicator use highlight a critical need for future research to develop a universally accepted framework for measuring ED overcrowding. Further studies should also explore the integration of input, throughput, and output indicators in predictive models to provide a more holistic understanding of patient flow dynamics.

From a policy perspective, the evidence suggests that interventions targeting diagnostic delays, staffing adequacy, and bed availability should be prioritized to reduce bottlenecks and improve patient outcomes. Policymakers and health care administrators may consider adopting composite measures, rather than relying on a single indicator, to guide resource allocation and performance evaluation. At the national level, embedding standardized overcrowding metrics into hospital accreditation and reporting systems could enhance comparability across institutions and support evidence-based policy development.

Overall, addressing ED overcrowding requires coordinated efforts at both institutional and policy levels. By strengthening measurement frameworks and aligning them with local contexts, health care systems can not only improve patient satisfaction and safety but also build greater resilience in responding to increasing health care demands.

## Supplementary material

10.2196/78073 Multimedia Appendix 1 Summary table of the result from search strategy for indicators.

10.2196/78073 Multimedia Appendix 2 List of indicators.

10.2196/78073 Checklist 1 PRISMA-ScR checklist.

## Abbreviations

ED: emergency department
ED LOS: emergency department length of stay
LWBS: left without being seen
OR: odds ratio
PRISMA-ScR: Preferred Reporting Items for Systematic Review and Meta-Analyses extension for Scoping Reviews
