# Supplementary figures and images for: Overcrowding Indicators in Emergency Departments Across Countries: Scoping Review
Source: Interact J Med Res. 2026 May 5;15:e78073. doi: 10.2196/78073 (PMC13143202; doi:10.2196/78073)

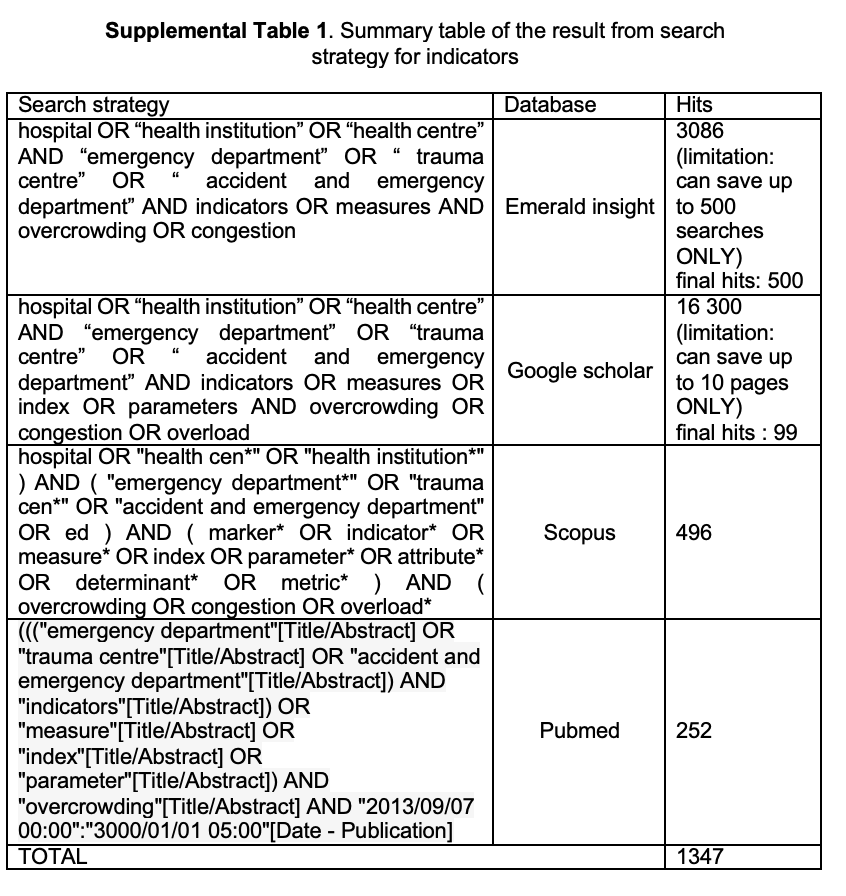

Supplement: Multimedia Appendix 1 [file ijmr-v15-e78073-s001.png]
