# Supplementary material for: Overcrowding Indicators in Emergency Departments Across Countries: Scoping Review
Source: Interact J Med Res. 2026 May 5;15:e78073. doi: 10.2196/78073 (PMC13143202; doi:10.2196/78073)
Supplement: Multimedia Appendix 2 [file ijmr-v15-e78073-s002.pdf]

APPENDICES

| No | List of authors | Study Title | Year of Publication | Study Design | Indicators | Any other specific tools to measure overcrowding (i.e NEDOCS, EDWIN) |
|---|---|---|---|---|---|---|
| 1. | Marsilio et al. [92] | Operations management solutions to improve ED patient flows: evidence from the Italian NHS | 2022 | Mixed-method | Length of stay (LOS)<br>Number Of Admissions<br>Number Of Doctors Per Admission<br>Number Of Nurses Per Admission<br>Skill Mix (amount of doctors / number of nurses) | Nil |
| 2. | Gorski et al. [44] | Crowding is the strongest predictor of left without being seen risk in a pediatric emergency department | 2021 | Retrospective Observational Study | Occupancy Rate (number of patients in treatment areas divided by the number of licensed ED beds)<br>Number of admitted patients (indexed to the total number of hospital beds)<br>Longest Admission Wait Time<br>Longest Waiting Room Wait Time<br>Left Without Being Seen (LWBS) | NEDOCS index (National ED Overcrowding Study Index) |
| 3. | Di Somma et al. [18] | Overcrowding in emergency department: an international issue | 2014 | Editorial | Length of Stay (LOS)<br>Patient Throughput Time<br>Patient Throughput<br>ED patients must be seen and then admitted or discharged within 4 hours presentation (4 hours rule) - target 98%, then 95% | Nil |

| No | List of authors | Study Title | Year of Publication | Study Design | Indicators | Any other specific tools to measure overcrowding (i.e NEDOCS, EDWIN) |
|---|---|---|---|---|---|---|
| | | | | | Number of patients waiting more than 8 hours for inpatient bed | |
| | | | | | Time to Initial Nursing Review | |
| | | | | | Time to Treatment | |
| | | | | | Patients Leaving The Department Without Being Seen | |
| | | | | | Service Experience | |
| | | | | | Hospital Occupancy Rate | |
| | | | | | Boarded Patient Time (The Process for implementing the transfer; max 2 patients / hallway) | |
| 4. | Scott et al. [62] | Deconstructing the 4-h rule for access to emergency care and putting patients first | 2014 | Review Article | Percentage of patients exiting ED within 4 hours of presentation | Nil |
| | | | | | Hospital Bed Occupancy | |
| | | | | | Number of ED attendances over the previous 24 hours | |
| | | | | | Boarding Time | |
| | | | | | Proportion of boarding patients in ED at a given time point | |
| 5. | Doan et al. [32] | The impact of pediatric emergency department crowding on patient and health care system outcomes: a | 2019 | Cohort | Length Of Stay (LOS) | Nil |
| | | | | | Leave Without Being Seen (LWBS) | |

| No | List of authors | Study Title | Year of Publication | Study Design | Indicators | Any other specific tools to measure overcrowding (i.e NEDOCS, EDWIN) |
|---|---|---|---|---|---|---|
| | | multicentre cohort study | | | | |
| 6. | Bucci et al. [67] | Emergency Department crowding and hospital bed shortage: is Lean a smart answer? A systematic review | 2016 | Systematic Review | Length Of Stay (LOS)<br>Left Without Being Seen (LWBS) | Nil |
| 7. | Van Der Linden et al. [7] | A different crowd, a different crowding level? The predefined thresholds of crowding scales may not be optimal for all emergency departments | 2018 | Cohort | Length Of Stay (LOS)<br>Total Patients in ED<br>Calculated Longest Admit Boarding Time (in ED)<br>Calculated Hours Longest Wait In WR (Last Bed Time) | Modified National ED OverCrowding Score (NEDOCS) |
| 8. | Otto et al. [93] | Length of stay as quality indicator in emergency departments: analysis of determinants in the German Emergency Department Data Registry (AKTIN registry) | 2022 | Cross-sectional observational study | Length Of Stay (LOS) | Nil |

| No | List of authors | Study Title | Year of Publication | Study Design | Indicators | Any other specific tools to measure overcrowding (i.e NEDOCS, EDWIN) |
|---|---|---|---|---|---|---|
| 9. | Li et al. [74] | A review on ambulance offload delay literature | 2018 | Review | Length Of Stay (LOS) | Nil |
| 10. | Hoot et al. [89] | Does crowding influence emergency department treatment time and disposition? | 2020 | Cohort | Treatment Time<br>ED Disposition | National Emergency Department Overcrowding Scale (NEDOCS) |
| 11. | Rahmani et al. [61] | Evaluation of overcrowding of emergency Department in Imam Reza Hospital in 2015 by implementing 2 scales: NEDOCS and EDWIN | 2017 | Cross-sectional Study | ED length of stay<br>EC occupancy rate | National Emergency Department Overcrowding Scale (NEDOCS)<br>Emergency Department Work Index (EDWIN) |
| 12. | Aksel et al. [19] | Effects of fast-track in a university emergency department through the National Emergency Department Overcrowding Study | 2014 | Cross-sectional Study | Length Of Stays<br>Left Without Being Seen | National Emergency Department Overcrowding Study (NEDOCS) |
| 13. | Fuentes et al. [68] | One-way-street' streamlined admission of critically ill trauma patients reduces emergency | 2016 | Case Control | Length Of Stay (LOS)<br>ICU Length Of Stay (LOS)<br>Hospital Length Of Stay (LOS) | Nil |

| No | List of authors | Study Title | Year of Publication | Study Design | Indicators | Any other specific tools to measure overcrowding (i.e NEDOCS, EDWIN) |
|---|---|---|---|---|---|---|
| | | department length of stay | | | | |
| 14. | Savioli et al. [114] | Five Level Triage vs. Four Level Triage in a Quaternary Emergency Department: National Analysis on Waiting Time, Validity, and Crowding-The CREONTE (Crowding and Re-Organization National TriagE) Study Group | 2023 | Observational | Wait Times<br>Number Of Patients Visiting The ED<br>Number Of People Who Left Without Being Seen (LWBS)<br>Length Of Stay (LOS)<br>Mean Number Or Percentage Of Admissions<br>Patients In The ED (Number Or Percentage)<br>Access Block And Boarding (Mean Number Or Percentage Of Patients Who Have Experienced It) | Nil |
| 15. | Kegel et al. [45] | The Impact of Extreme Heat Events on Emergency Departments in Canadian Hospitals | 2021 | Cohort | Length Of Stay (LOS) | Nil |
| 16. | Van Der Linden et al. [117] | Effects of process changes on emergency department crowding in a changing world: an interrupted time - series analysis | 2023 | Mixed Methods | Length Of Stay (LOS)<br>Number Of Patients Experiencing Exit Block | Modified version of the National ED Overcrowding Scale (MNEDOCS) |
| 17. | Philips et al. [53] | Overcrowding and its association with | 2017 | Cohort | Length Of Stay (LOS) | National Emergency Department |

| No | List of authors | Study Title | Year of Publication | Study Design | Indicators | Any other specific tools to measure overcrowding (i.e NEDOCS, EDWIN) |
|---|---|---|---|---|---|---|
| | | patient outcomes in a median-low volume emergency department | | | | Overcrowding Score (NEDOCS)<br>Community Emergency Department Overcrowding Score (CEDOCS)<br>Severely overcrowded – Overcrowded–Not overcrowded Estimation Tool (SONET) |
| 18. | Casalino et al. [11] | Predictive variables of an emergency department quality and performance indicator: a 1 year prospective, observational, cohort study evaluating hospital and emergency census variables and emergency department time interval measurements | 2013 | Cohort | Length Of Stay (LOS)<br>Wait Time To Triage Nurse<br>Wait Time To Provider (ED Physician)<br>Time Interval from ED Arrival To Decision<br>Time Interval From Decision Time To Departure Time<br>Percentage Of Patients Leaving The ED In Less Than 4 hours | Nil |
| 19. | Tekwani at al. [112] | Emergency department crowding is associated with reduced satisfaction scores in patients discharged from the | 2013 | Cohort | ED Occupancy Rate<br>Hospital Diversion Status | Modified Emergency Department Work Index (mEDWIN) |

| No | List of authors | Study Title | Year of Publication | Study Design | Indicators | Any other specific tools to measure overcrowding (i.e NEDOCS, EDWIN) |
|---|---|---|---|---|---|---|
| | | emergency department | | | | |
| 20. | Lee et al. [105] | Emergency department overcrowding and ambulance turnaround time | 2015 | Cross-sectional Study<br>I | Average Occupancy Rate<br>Ambulance Turnaround Time | Nil |
| 21. | Georgio et al. [58] | Emergency Department Flow Measures for Adult and Pediatric Patients in British Columbia and Ontario: A Retrospective, Repeated Cross-Sectional Study | 2017 | Retrospective repeated Cross-sectional study | Wait Time (WT) to see a physician<br>Length Of Stay (LOS)<br>The Proportion Of Patients Leaving Without Being Seen (LWBS)<br>Admission Rates<br>Canadian Triage and Acuity Scale (CTAS) | Nil |
| 22. | Patel et al. [17] | Reduction of admit wait times: the effect of a leadership-based program | 2014 | Quasi-experimental | Percentage of patient admitted within 60 minutes<br>Boarding Time Per Admission<br>Lengths Of Stay (LOS) for admitted patients, discharged patients, and all patients<br>Leave Without Being Seen (LWBS)<br>Ambulance Diversion Hours<br>Patient Satisfaction Scores | Nil |

| No | List of authors | Study Title | Year of Publication | Study Design | Indicators | Any other specific tools to measure overcrowding (i.e NEDOCS, EDWIN) |
|---|---|---|---|---|---|---|
| 23. | Tseng et al. [94] | Examining patient flow in a tertiary hospital's emergency department at a low coronavirus prevalence region | 2022 | Descriptive Cross-sectional Observational Study | Length Of Stay (LOS) | Nil |
| 24. | Li et al. [69] | Influence of CT utilisation on patient flow in the emergency department: a retrospective 1 year cohort study | 2016 | Retrospective Cohort Study | ED Length Of Stay | Nil |
| 25. | Truong et al. [57] | Emergency Department Return Visits Within a Large Geographic Area | 2017 | Retrospective Observational Study | Daily Average Length Of Stay (LOS)<br>The daily proportion of patients who left without being seen by a physician (LWBS)<br>The total daily volume of visits<br>The number of admitted patients boarding in the ED at noon and at midnight at the site | Nil |
| 26. | d’Etienne et al. [82] | Two-step predictive model for early detection of emergency department patients with prolonged stay | 2020 | Retrospective Observational Study | Patient ED Length Of Stay (LOS) | National ED Overcrowding Scale (NEDOCS) |

| No | List of authors | Study Title | Year of Publication | Study Design | Indicators | Any other specific tools to measure overcrowding (i.e NEDOCS, EDWIN) |
|---|---|---|---|---|---|---|
| | | and its management implications | | | | |
| 27. | Wretborn et al. [100] | Differentiating properties of occupancy rate and workload to estimate crowding: A Swedish national cross-sectional study | 2022 | Observational Cross-sectional Study | Occupancy Rate | Skåne Emergency Department Assessment of Patient Load (SEAL) |
| | | | | | Time waiting for a physician is the time from triage and nursing procedures to first physician contact | National Emergency Department Overcrowding Score (NEDOCS) |
| | | | | | | Simplified International Crowding Measure in Emergency Department (SICMED) |
| 28. | Xu and Ho [28] | Freestanding emergency departments in Texas do not alleviate congestion in hospital-based emergency departments | 2019 | Retrospective Cohort Study | Wait Time | Nil |
| | | | | | Length of visit for discharged patients | |
| | | | | | Rate of patients who Left Without Being Seen (LWBS) | |
| | | | | | Total number of visits to hospital ED | |
| | | | | | Average Hospital-Based ED Visit | |
| | | | | | The annual number of visits to hospital ED | |
| | | | | | Discharge Time | |
| | | | | | Visit Volume | |
| 29. | Forero et al. [31] | Impact of the four-hour National Emergency Access | 2019 | Quasi-Experimental Study | 'ED flow' as the percentage of presentations with an ED length of stay | Nil |

| No | List of authors | Study Title | Year of Publication | Study Design | Indicators | Any other specific tools to measure overcrowding (i.e NEDOCS, EDWIN) |
|---|---|---|---|---|---|---|
| | | Target on 30 day mortality, access block and chronic emergency department overcrowding in Australian emergency departments | | | (EDLOS) within 4 hours | |
| | | | | | Access Block | |
| | | | | | Admitted Patient 30 Days Mortality Rate | |
| 30. | Al-Qahtani et al. [46] | Exploring Potential Association Between Emergency Department Crowding Status and Patients' Length of Stay at a University Hospital in Saudi Arabia | 2021 | Retrospective Cohort Analysis Study | ED Occupancy Rates | Nil |
| | | | | | Length Of Stay (LOS) in ED | |
| | | | | | Percentage Of Patient Who Spent In ED More Than 6 Hours | |
| 31. | Theodoro et al. [51] | Central venous catheter adverse events are not associated with crowding indicators | 2021 | Retrospective Observational Study | Number Of Patients In The Waiting Room (WR) | National Emergency Department Overcrowding Scale (NEDOCS) |
| | | | | | Number Of Admitted Patients In The ED Awaiting Inpatient Beds Or “Boarders” | |
| | | | | | ED Occupancy (EDO) | |
| 32. | Dai and Shi [63] | A two-time-scale approach to time-varying queues in | 2017 | Interventional Study | ED Boarding Time | Nil |
| | | | | | Length Of Stay (LOS) | |
| | | | | | Departure Time | |

| No | List of authors | Study Title | Year of Publication | Study Design | Indicators | Any other specific tools to measure overcrowding (i.e NEDOCS, EDWIN) |
|---|---|---|---|---|---|---|
| | | hospital inpatient flow management | | | | |
| 33. | Street et al. [56] | Influences on emergency department length of stay for older people | 2017 | Retrospective Cohort Study | ED Length Of Stay<br>The proportion of ED patients discharged within 4 hours (measure of overcrowding)<br>The proportion of admitted patients leaving ED within 8 hours (measure of access block) | Nil |
| 34. | Van Der Linden et al. [33] | The impact of a multimodal intervention on emergency department crowding and patient flow | 2019 | Cross-Sectional Study | Radiology Turnaround Times<br>Patients' Length Of Stay (LOS)<br>Proportion of patients leaving without being seen (LWBS) by a medical provider | Nil |
| 35. | Shah et al. [83] | Impact of Provider-In-Triage in a Safety-Net Hospital | 2020 | Quasi-Experimental Study | ED Length Of Stay (EDLOS)<br>Patients Leaving Without Being Seen (LWBS)<br>Door-To-Doctor (DTD)<br>Left Without Treatment (LWOT) | Nil |
| 36. | Kadri et al. [95] | Towards accurate prediction of patient length of stay at emergency department: a GAN-driven deep learning framework | 2022 | Retrospective Observational Study | Length Of Stay (LOS) | Nil |

| No | List of authors | Study Title | Year of Publication | Study Design | Indicators | Any other specific tools to measure overcrowding (i.e NEDOCS, EDWIN) |
|---|---|---|---|---|---|---|
| 37. | Badr et al. [96] | Measures of Emergency Department Crowding, a Systematic Review. How to Make Sense of a Long List | 2022 | Systematic review | ED Occupancy | National ED Overcrowding Scale (NEDOCS) |
| | | | | | ED Length Of Stay | Emergency Department Work Index (EDWIN) |
| | | | | | ED Volume | |
| | | | | | ED Boarding Time | |
| | | | | | Number of Boarders | |
| | | | | | Waiting Room Number | |
| 38. | Kim et al. [81] | Influence of Overcrowding in the Emergency Department on Return Visit within 72 Hours | 2020 | Retrospective Observational Study | Boarding patients during the first 1 hour from ED arrival time | Nil |
| | | | | | Boarding patients during the first 4 hours from ED arrival time | |
| | | | | | Boarding patients the last 1 hour before ED departure | |
| | | | | | The number of total patients occupying the ED | |
| | | | | | The number of evaluating patients to estimate the overload in the throughput of the ED | |
| | | | | | The number of boarding patients to reflect the blocking of ED output | |
| 39. | Santos et al. [64] | The effects of emergency department overcrowding on admitted patient outcomes: a | 2016 | Systematic review protocol | Length Of Stay (LOS) in the ED | Nil |
| | | | | | The number of patients admitted to the ED | |

| No | List of authors | Study Title | Year of Publication | Study Design | Indicators | Any other specific tools to measure overcrowding (i.e NEDOCS, EDWIN) |
|---|---|---|---|---|---|---|
| | | systematic review protocol | | | | |
| 40. | Peng et al. [85] | Evaluation of physician in triage impact on overcrowding in emergency department using discrete-event simulation | 2020 | Cross-Sectional Study | Length Of Stay in the ED<br>Patient waiting times to see a doctor (i.e. WTBS)<br>Patients Leaving Without Being Seen (LWBS) | Nil |
| 41. | Weiss et al. [16] | Evaluating community ED crowding: the Community ED Overcrowding Scale study | 2014 | Cross-Sectional Study | ED visits / year number of ED visits/year as presented to State Health Planning Office<br>No. of ED beds number of ED beds as presented to State Health Planning Office<br>Number of hospital beds: Number hospital beds as presented to State Health Planning Office<br>Total patients in the ED<br>Total number of requested hospital admissions from the ED<br>Longest admission time and waiting room time for the last patient called | National ED Overcrowding Scale (NEDOCS), Community Emergency Department Overcrowding Score (CEDOCS) |
| 42. | Strada et al. [29] | Do health care professionals' perceptions help to measure the degree | 2019 | Cross-Sectional Observational Study | Total patients in the ED<br>Total number of requested hospital admissions from the ED | National ED Overcrowding Scale (NEDOCS) |

| No | List of authors | Study Title | Year of Publication | Study Design | Indicators | Any other specific tools to measure overcrowding (i.e NEDOCS, EDWIN) |
|---|---|---|---|---|---|---|
| | | of overcrowding in the emergency department? A pilot study in an Italian University hospital | | | Longest admission time and waiting room time for the last patient called. | |
| 43. | Khanna et al. [26] | Using capacity alert calls to reduce overcrowding in a major public hospital | 2014 | Cross-Sectional Study | Bed Occupancy (%)<br>Average access block (admitted patient's boarding time in ED more 8 hours)<br>Average discharge rate (patients/hr)<br>Average admission rate (patients/hour) | Nil |
| 44. | Doan et al. [20] | Trends in use in a Canadian pediatric emergency department | 2014 | Retrospective Cohort Study | Patients leaving without being seen (LWBS)<br>Length Of Stay (LOS) | Nil |
| 45. | Ngo et al. [73] | Impact of the Four-Hour Rule in Western Australian hospitals: Trend analysis of a large record linkage study 2002-2013 | 2018 | Intervention study | ED Occupancy Rate<br>ED Length of Stay (LOS)<br>Time to being seen by ED clinician<br>Access Block (defined as ED length of stay (ED LOS) longer than 8 hours) for an admitted patient<br>ED attendances and re-attendance (within ED attendances and re-attendance (within seven days of index ED discharge) | Nil |

| No | List of authors | Study Title | Year of Publication | Study Design | Indicators | Any other specific tools to measure overcrowding (i.e NEDOCS, EDWIN) |
|---|---|---|---|---|---|---|
| | | | | | Did Not Wait (DNW) - left ED before being seen by an ED clinician | |
| | | | | | Admission Rate | |
| 46. | Burke et al. [55] | Two Hour Evaluation and Referral Model for Shorter Turnaround Times in the emergency department | 2017 | Prospective Observational Study | ED length of stay (LOS) | Nil |
| | | | | | Wait times by triage category | |
| | | | | | Ambulance offload times (triage to first clinical location) | |
| | | | | | Average time to referral | |
| | | | | | ED workup time (triage to departure ready) | |
| | | | | | Bed delay (bed request to actual departure) | |
| 47. | Boyle et al. [70] | Comparison of the International Crowding Measure in Emergency Departments (ICMED) and the National Emergency Department Overcrowding Score (NEDOCS) to measure emergency department crowding: pilot study | 2016 | Cross-Sectional Study | Patients who leave without being seen or treated (LWBS) | Nil |
| | | | | | Time until triage | |
| | | | | | ED occupancy rate | |
| | | | | | Patients' total length of stay in ED | |
| | | | | | Time until a physician first sees the patient | |
| | | | | | ED boarding time | |
| | | | | | Number of patients boarding in the ED | |
| 48. | Watson and Stuart [115] | Improving Safety and Quality With an Emergency | 2023 | Quality Improvement | Median LOS | Nil |
| | | | | | left without being seen (LWBS) | |

| No | List of authors | Study Title | Year of Publication | Study Design | Indicators | Any other specific tools to measure overcrowding (i.e NEDOCS, EDWIN) |
|---|---|---|---|---|---|---|
| | | Department Overcrowding Plan | | | Median time from decision to admit to completion of patient admission | |
| | | | | | Median X-ray turnaround time for ED patients | |
| | | | | | Median computed tomography scan turnaround time for ED patients | |
| 49. | Erenler et al. [15] | Reasons for overcrowding in the emergency department: experiences and suggestions of an education and research hospital | 2014 | Cross-Sectional Study | mean LOS Length of stay in observation room of ED | Nil |
| | | | | | Annual ED admission count, seasonal distribution | |
| | | | | | Number of repeated visits within 24 hours | |
| 50. | Thapa et al. [75] | Application of RFID technology to reduce overcrowding in hospital emergency departments | 2018 | Quasi Experimental | ED LOS | |
| | | | | | ED wait times | |
| 51. | Vegting et al. [111] | What are we waiting for? Factors influencing completion times in an academic and peripheral emergency department | 2015 | Observational Study | Average LOS per age | Nil |
| | | | | | Door-to-doctor time | |
| | | | | | Patient completion time within 4hrs (%) | |
| 52. | Menon et al. [41] | Bed Utilization and Overcrowding in a | 2021 | Prospective Observational Study | Daily bed occupancy rate | |
| | | | | | ED LOS | |

| No | List of authors | Study Title | Year of Publication | Study Design | Indicators | Any other specific tools to measure overcrowding (i.e NEDOCS, EDWIN) |
|---|---|---|---|---|---|---|
| | | High-Volume Tertiary Level Pediatric Emergency Department | | | Discontinued care and left against medical advice (LAMA)<br>number of boarders (median)<br>total number of ED visits | |
| 53. | Chiu et al. [60] | The influence of crowding on clinical practice in the emergency department | 2017 | Retrospective Cohort | Decision making time of EP<br>ED Length Of Stay (LOS) | Nil |
| 54. | de Araujo et al. [103] | Does overcrowding and health insurance type impact patient outcomes in emergency departments? | 2013 | Cross-Sectional Study | Wait time before seeing a doctor<br>Average number of patients in the ED at the time the patient checks | Nil |
| 55. | Konrad et al. [107] | Modeling the impact of changing patient flow processes in an emergency department: Insights from a computer simulation study | 2013 | Simulation Study | Door to doctor time<br>ED Length Of Stay (LOS) | Nil |
| 56. | Elalouf and Wachtel [108] | An alternative scheduling approach for improving patient-flow in emergency departments | 2015 | Case Study | ED Length Of Stay (LOS) | Nil |

| No | List of authors | Study Title | Year of Publication | Study Design | Indicators | Any other specific tools to measure overcrowding (i.e NEDOCS, EDWIN) |
|---|---|---|---|---|---|---|
| 57. | Ming et al. [65] | Can Team Triage Improve Patient Flow in the Emergency Department? A Systematic Review and Meta-Analysis | 2016 | Systematic Review | ED Length Of Stay (LOS)<br>Patient disappearance<br>Waiting Time (time to first physician assessment)<br>Reattendance (as adverse outcome)<br>Disappearance (LWBS) | Nil |
| 58. | Ghanes et al. [71] | Modeling and analysis of triage nurse ordering in emergency departments | 2016 | Case Study | ED Length Of Stay (LOS) | Nil |
| 59. | Spencer et al. [34] | Health Care Provider in Triage to Improve Outcomes | 2019 | Quality Improvement | Length Of Stay (LOS)<br>Left Without Being Seen (LWBS) Rate<br>Patient satisfaction<br>Door to provider time<br>Door to disposition times (admit and discharge) | Nil |
| 60. | Ajmi et al. [30] | Agent-based dynamic optimization for managing the workflow of the patient's pathway | 2019 | Observational / Simulation | Ambulances Reorientation<br>Length Of Stay (LOS)<br>First Consultation Time (FCT)<br>Drop Out Patients (DOP) | Nil |
| 61. | Kenny et al. [47] | Patient flow simulation using | 2021 | Simulation Study | Patient Waiting Time<br>Length Of Stay (LOS) | Nil |

| No | List of authors | Study Title | Year of Publication | Study Design | Indicators | Any other specific tools to measure overcrowding (i.e NEDOCS, EDWIN) |
|---|---|---|---|---|---|---|
| | | historically informed synthetic data | | | | |
| 62. | Del Torto et al. [37] | Length of stay reduction in the emergency department and its quantification using complex network theory | 2019 | Case Study | Mean Length Of Stay (LOS) according to color triaging | Nil |
| 63. | Noris et al. [97] | Lean Healthcare Implementation in Malaysian Specialist Hospitals: Challenges and Performance Evaluation | 2022 | Quality Improvement | ED Length Of Stay (LOS) | Nil |
| | | | | | Bed Waiting Time | |
| | | | | | Arrival to Consultation | |
| | | | | | Discharge time | |
| 64. | Le et al. [50] | Lean management for improving hospital waiting times-Case study of a Vietnamese public / general hospital emergency department | 2021 | Quantitative Study With Pre- And Post-Lean Design | Waiting time for medical procedures / operations | Nil |
| | | | | | Waiting time for cardiac intervention | |
| | | | | | Waiting time to be assessed by physicians | |
| | | | | | Waiting time for transfer to other clinical departments | |
| 65. | Wretborn et al. [49] | Validation of the modified Skåne emergency department assessment of patient load (mSEAL) model for | 2021 | Observational Cross Sectional Study | Occupancy Rate. | Skåne Emergency Department Assessment of Patient Load (SEAL) |
| | | | | | Time To Physician | Simplified International Crowding Measure in Emergency Department (SICMED) |

| No | List of authors | Study Title | Year of Publication | Study Design | Indicators | Any other specific tools to measure overcrowding (i.e NEDOCS, EDWIN) |
|---|---|---|---|---|---|---|
| | | emergency department crowding and comparison with international models; an observational study. | | | | |
| 66. | Molla et al. [80] | A Lean Six Sigma Quality Improvement Project Improves Timeliness of Discharge from the Hospital | 2018 | Quality Improvement Project | 30 days readmission rates | Nil |
| 67. | Davis et al. [40] | Emergency department resilience to disaster level overcrowding: a component resilience framework for analysis and predictive modeling | 2019 | Simulation And Predictive Modeling Study | Number of patients waiting on an inpatient bed | National ED Overcrowding Scale (NEDOCS) |
| | | | | | Longest boarding time of patients in the ED waiting to be admitted to the hospital | |
| 68. | Wang et al. [104] | Use of the SONET score to evaluate high volume emergency department overcrowding: a prospective derivation and validation study | 2015 | Prospective Study | Left Without Being Seen (LWBS) | Severely overcrowded-Overcrowded-Not overcrowded Estimation Tool (SONET) |
| | | | | | Average Length Of Stay (LOS) | Total Emergency Severity Index (TESI) |
| | | | | | ED 72 hours returns<br>Mortality Rate | National ED Overcrowding Scale (NEDOCS) |
| 69. | Hsu et al. [3] | Emergency department | 2019 | Quality Improvement Project | Length Of Stay (LOS) | Nil |

| No | List of authors | Study Title | Year of Publication | Study Design | Indicators | Any other specific tools to measure overcrowding (i.e NEDOCS, EDWIN) |
|---|---|---|---|---|---|---|
| | | overcrowding: Quality improvement in a Taiwan Medical Center | | | | |
| 70. | Acuna et al. [39] | Ambulance allocation optimization model for the overcrowding problem in US emergency departments: A case study in Florida | 2019 | Case Study | Mean time to treatment<br>Average ED waiting time | Nil |
| 71. | Handel et al. [22] | Association of emergency department and hospital characteristics with elopements and length of stay | 2014 | Cross-Sectional Study | Annual ED Volume<br>Percentage of patients admitted<br>Percentage of patients presenting by ambulance<br>Median Length Of Stay (LOS)<br>Left Before Treatment Is Complete (LBTC) | Nil |
| 72. | Khubrani et al. [90] | Association between emergency department overcrowding and mortality at a teaching hospital in Saudi Arabia | 2020 | Cross-Sectional Study | ED Occupancy Rate<br>Mortality Rate | Nil |
| 73. | Berg et al. [35] | Associations between crowding and ten-day mortality | 2019 | Retrospective cohort study | ED mean Length Of Stay (LOS)<br>ED Occupancy Ratio | Nil |

| No | List of authors | Study Title | Year of Publication | Study Design | Indicators | Any other specific tools to measure overcrowding (i.e NEDOCS, EDWIN) |
|---|---|---|---|---|---|---|
| | | among patients allocated lower triage acuity levels without need of acute hospital care on departure from the emergency department | | | Mortality within 10 days for the group of patients with triage acuity levels 3 to 5 and without need of acute hospital care on departure from the ED | |
| 74. | Improta et al. [99] | A case study to investigate the impact of overcrowding indices in emergency departments | 2022 | Case Study | Patient Admission | Emergency Department Work Index (EDWIN)<br>National ED Overcrowding Scale (NEDOCS) |
| 75. | Soares [52] | Analysis of the Internal Bed Regulation Committees from hospitals of a Southern Brazilian city | 2017 | Cross-Sectional Study | Hospital Occupancy Rate<br>Mean ED Length Of Stay (LOS)<br>Rate of patients with prolonged stay<br>Patients waiting for external transfer<br>Production (Number of admissions, surgeries, appointments)<br>Duration of patient flow throughout hospital facilities<br>Patients Day<br>Renewal Rate (bed turnover)<br>Bed Turnover Interval | Nil |
| 76. | Hsieh et al. [116] | Implementation of Vertical Split Flow | 2023 | Retrospective Cohort Study | ED Length Of Stay (LOS) | Nil |

| No | List of authors | Study Title | Year of Publication | Study Design | Indicators | Any other specific tools to measure overcrowding (i.e NEDOCS, EDWIN) |
|---|---|---|---|---|---|---|
| | | Model for Patient Throughput at a Community Hospital Emergency Department | | | | |
| 77. | Burström et al. [66] | Improved quality and efficiency after the introduction of physician-led team triage in an emergency department | 2016 | Retrospective Study | Time to physician | Nil |
| | | | | | Time from physician to discharge | |
| | | | | | Length Of Stay (LOS) | |
| | | | | | 4 hours Turnover Rate | |
| | | | | | Left without being seen or treatment not completed | |
| | | | | | 24 hours unscheduled return (quality outcome variable) | |
| | | | | | 72 hours unscheduled return (quality outcome variable) | |
| | | | | | 7 days mortality (quality outcome variable) | |
| | | | | | 30 days mortality (quality outcome variable) | |
| 78. | Lin et al. [106] | Managing emergency department overcrowding via ambulance diversion: a discrete event simulation model | 2015 | Discrete Event Simulation Study | Patient waiting time for service | Crowdedness index (CI) |
| | | | | | Average percentage of adverse patients | |
| 79. | Hsu et al. [118] | Why do general medical patients have a lengthy wait | 2014 | Retrospective Study | ED Length Of Stay (LOS) | Nil |

| No | List of authors | Study Title | Year of Publication | Study Design | Indicators | Any other specific tools to measure overcrowding (i.e NEDOCS, EDWIN) |
|---|---|---|---|---|---|---|
| | | in the emergency department before admission? | | | | |
| 80. | Hofer and Saurenmann [119] | Parameters affecting length of stay in a pediatric emergency department: a retrospective observational study | 2017 | Retrospective Observational Study | ED Length Of Stay (LOS) | Nil |
| 81. | Kim et al. [109] | Improving process quality for pediatric emergency department | 2013 | Retrospective Case Study | ED Length Of Stay (LOS) | Nil |
| 82. | Schmutz et al. [120] | No waiting lying in a corridor: a quality improvement initiative in an emergency department | 2023 | Quality Improvement Initiative | Waiting time before medical care | Nil |
| | | | | | Waiting time before nurse triage | |
| 83. | Sullivan et al. [23] | Aiming to be NEAT: safely improving and sustaining access to emergency care in a tertiary referral hospital | 2014 | Quality Improvement Study | Percentage of patients exiting the ED within 4 hours | Nil |
| | | | | | Total mean transit time from ED presentation to discharge | |
| | | | | | ‘Did Not Wait’ rates in ED | |
| 84. | Van Der Linden et al. [101] | Emergency department crowding in the Netherlands: Managers' experiences | 2013 | Survey | Mean annual ED visits | Nil |
| | | | | | Mean Length Of Stay (LOS) for discharged patients | |
| | | | | | Mean Length Of Stay (LOS) for admitted patients | |

| No | List of authors | Study Title | Year of Publication | Study Design | Indicators | Any other specific tools to measure overcrowding (i.e NEDOCS, EDWIN) |
|---|---|---|---|---|---|---|
| 85. | Tsai et al. [88] | Adjusting daily inpatient bed allocation to smooth emergency department occupancy variation | 2020 | Discrete Event Simulation | Proportion of patients held >24 hours<br>Proportion of patients held >48 hours<br>Proportion of admitted patients held >24 hours<br>Proportion of admitted patients held >48 hours | Nil |
| 86. | Trotzky et al. [48] | Do automatic push notifications improve patient flow in the emergency department? analysis of an ED in a large medical center in Israel | 2021 | Prospective Cohort Study | Total Length Of Stay (LOS)<br>Time to Triage<br>Time to first MD<br>Time from Triage to first MD<br>Time to decision | Nil |
| 87. | Mallows [98] | Effects of staff grade, overcrowding and presentations on emergency department performance: A regression model | 2022 | Cross-Sectional Study | Daily Emergency Treatment Performance (ETP) for all ED patients (total ETP)<br>Patients admitted to the ward (admit ETP)<br>patients admitted to the Emergency Medicine Short Stay (EMSS ETP)<br>Discharged patients (discharge ETP)<br>Daily median waiting time<br>Daily percentage of patients who left at own risk (LOR) | Nil |

| No | List of authors | Study Title | Year of Publication | Study Design | Indicators | Any other specific tools to measure overcrowding (i.e NEDOCS, EDWIN) |
|---|---|---|---|---|---|---|
| | | | | | Daily triage category performance for triage categories 2–5 | |
| | | | | | The number of admitted patients boarded in the ED waiting for a ward bed at 8 am | |
| 88. | Cirillo et al. [42] | Impact of emergency short-stay unit opening on in-hospital global and cardiology indicators | 2021 | Retrospective Observational Study | Number of ED visits | Nil |
| | | | | | Average daily patients in ED beds | |
| | | | | | ED Bed Occupancy Rate (BOR) | |
| | | | | | Number of total in-hospital admissions | |
| | | | | | Number of in-hospital admissions from ED and not from ED (elective admissions) | |
| | | | | | Number of in-hospital admissions from ED at Short Stay Unit (SSU) | |
| | | | | | Percent admissions from ED | |
| | | | | | Percent admissions at Short Stay Unit (SSU) | |
| | | | | | Length Of Stay (LOS) | |
| 89. | Wang et al. [54] | Optimal Measurement Interval for Emergency Department | 2017 | Cohort study | ED Length Of Stay (LOS) | National Emergency Department Overcrowding Scale (NEDOCS) |
| | | | | | Left Without Being Seen (LWBS) | Severely Overcrowded, Overcrowded, and Not |

| No | List of authors | Study Title | Year of Publication | Study Design | Indicators | Any other specific tools to measure overcrowding (i.e NEDOCS, EDWIN) |
|---|---|---|---|---|---|---|
| | | Crowding Estimation Tools | | | | Overcrowded Estimation Tool (SONET) |
| | | | | | | Emergency Department Work Index (EDWIN) |
| 90. | Fayyaz et al. [110] | Missing the boat: Odds for the patients who leave ED without being seen | 2013 | Retrospective Patient Record Review | Length Of Stay (LOS) | Nil |
| | | | | | Waiting time before getting a bed (waiting after patient is assigned a bed in ED) | |
| | | | | | Percentages of left without being seen | |
| 91. | Korsten et al. [21] | Impact of professional quality management on interdisciplinary emergency care units | 2014 | Retrospective Observational Study | Length Of Stay (LOS) | Nil |
| | | | | | Diagnostic errors | |
| 92. | Andrews et al. [76] | Improving Patient Flows at St. Andrew's War Memorial Hospital's Emergency Department Through Process Mining | 2018 | Case Study | Length Of Stay (LOS) | Nil |
| 93. | Landa et al. [59] | A hybrid simulation approach to analyse patient boarding in emergency departments | 2017 | System Dynamics and Discrete Event Simulation Study | Length Of Stay (LOS) | Nil |
| 94. | | | 2016 | | Length Of Stay (LOS) | Nil |

| No | List of authors | Study Title | Year of Publication | Study Design | Indicators | Any other specific tools to measure overcrowding (i.e NEDOCS, EDWIN) |
|---|---|---|---|---|---|---|
| | Cocke et al. [72] | UVA emergency department patient flow simulation and analysis | | Discrete Event Simulation | Average arrival to provider time | |
| 95. | Wallingford et al. [77] | Introduction of a Horizontal and Vertical Split Flow Model of Emergency Department Patients as a Response to Overcrowding | 2018 | Retrospective Pre- And Post Intervention Study | Length Of Stay (LOS) | Nil |
| 96. | Salehi et al. [78] | Emergency department boarding: A descriptive analysis and measurement of impact on outcomes | 2018 | Retrospective Single-Centre Observational Study | Length Of Stay (LOS)<br>Boarding Time | Nil |
| 97. | Boyle et al. [36] | Coxian Phase-Type Regression Models for Understanding the Relationship Between Patient Attributes, Overcrowding, and Length of Stay (LOS) in Hospital Emergency Departments | 2019 | Retrospective Observational Study | Length Of Stay (LOS) | Ni |
| 98. | Van der Veen et al. [79] | Independent determinants of prolonged | 2018 | Prospective Observational Cohort Study | Length Of Stay (LOS) | Nil |

| No | List of authors | Study Title | Year of Publication | Study Design | Indicators | Any other specific tools to measure overcrowding (i.e NEDOCS, EDWIN) |
|---|---|---|---|---|---|---|
| | | emergency department length of stay in a tertiary care centre: A prospective cohort study | | | | |
| 99. | Savioli et al. [43] | How the coronavirus disease 2019 pandemic changed the patterns of healthcare utilization by geriatric patients and the crowding: a call to action for effective solutions to the access block | 2021 | Retrospective Observational Study | Waiting Time | Nil |
| | | | | | Process Time | |
| | | | | | Number of patients visiting the ED | |
| | | | | | Disease severity and complexity | |
| | | | | | Number of people who left without being seen | |
| | | | | | ED Length Of Stay (LOS) | |
| | | | | | Mean number or percent of admissions | |
| | | | | | Patients in the ED (number or percent) | |
| | | | | | Access block and boarding (the mean number or percent) | |
| | | | | | Access block or boarding times (total access block time) | |
| 100. | Choi et al. [86] | Admission Decisions Made by Emergency Physicians Can Reduce the Emergency Department Length of Stay for Medical Patients | 2020 | Retrospective Observational Study | Length Of Stay (LOS) | Nil |

| No | List of authors | Study Title | Year of Publication | Study Design | Indicators | Any other specific tools to measure overcrowding (i.e NEDOCS, EDWIN) |
|---|---|---|---|---|---|---|
| 101. | Payne et al. [87] | Improving Throughput for Mid-acuity Patients in the Pediatric Emergency Department | 2020 | Case Study | Time-to-first-provider for mid-acuity patients<br>ED Length Of Stay (LOS)<br>Time to first nursing assessment<br>Proportion of high acuity patients seen within 20 minutes | Nil |
| 102. | Santos et al. [64] | The effects of emergency department overcrowding on admitted patient outcomes: a systematic review protocol | 2016 | Systematic Review Protocol | Length Of Stay (LOS) | Nil |
| 103. | Wang et al. [24] | The inaccuracy of determining overcrowding status by using the national ED overcrowding study tool | 2014 | Cross-Sectional Observational Study | ED Length Of Stay (LOS) LOS<br>Number of patients left without being seen | National Emergency Department Overcrowding Scale (NEDOCS) |
| 104. | Jo et al. [25] | Emergency department occupancy ratio is associated with increased early mortality | 2014 | Retrospective Study | ED Length Of Stay (LOS) LOS<br>ED Occupancy Rate | Nil |
| 105. | Berg et al. [38] | Significant changes in emergency | 2018 | Retrospective observational study | ED Occupancy Ratio (EDOR)<br>ED Length Of Stay (LOS) | Nil |

| No | List of authors | Study Title | Year of Publication | Study Design | Indicators | Any other specific tools to measure overcrowding (i.e NEDOCS, EDWIN) |
|---|---|---|---|---|---|---|
| | | department length of stay and case mix over eight years at a large Swedish University Hospital | | | Registered nurse /physician per patient ratio<br>Patients per registered nurse / physician's ratio | |
| 106. | Kang et al. [113] | ED crowding and the outcomes of out-of-hospital cardiac arrest | 2015 | Retrospective study | ED Occupancy Rate | Nil |
| 107. | Johnson and Winkelman [102] | The effect of emergency department crowding on patient outcomes | 2015 | Literature review | Occupancy Rate<br>ED Length Of Stay (LOS)<br>Left Without Being Examined<br>Number of patients visits<br>Prolonged ED boarding stay<br>Ambulance diversion | Nil |
| 108. | Wachtel and Elalouf [84] | Addressing overcrowding in an emergency department: an approach for identifying and treating influential factors and a real-life application | 2020 | Case Study | ED length of stay | |
| 109. | Handel et al. [27] | Associations between patient and emergency department operational | 2014 | Retrospective observational study | ED length of stay<br>Average number patients visiting ED | |

| No | List of authors | Study Title | Year of Publication | Study Design | Indicators | Any other specific tools to measure overcrowding (i.e NEDOCS, EDWIN) |
|---|---|---|---|---|---|---|
| | | characteristics and patient satisfaction scores in an adult population | | | | |
| 110. | George et al. [121] | Effect of population ageing on emergency department speed and efficiency: a historical perspective from a district general hospital in the UK | 2006 | Retrospective observational study | Time to decision/referal | |
| 111. | Bélanger et al. [91] | Health Care Systems Engineering | 2020 | Scholarly Text | Mortality rate | |

## REFERENCE


3. Hsu CM, Liang LL, Chang YT, Juang WC. Emergency department overcrowding: quality improvement in a Taiwan Medical Center. J Formos Med Assoc. Jan 2019;118(1 Pt 1):186-193. [doi: ] [Medline: 29665984]

7. Van Der Linden MC, Van Loon M, Gaakeer MI, Richards JR, Derlet RW, Van Der Linden N. A different crowd, a different crowding level? The predefined thresholds of crowding scales may not be optimal for all emergency departments. Int Emerg Nurs. Nov 2018;41:25-30. [doi: ] [Medline: 29880260]

11. Casalino E, Choquet C, Bernard J, et al. Predictive variables of an emergency department quality and performance indicator: a 1-year prospective, observational, cohort study evaluating hospital and emergency census variables and emergency department time interval measurements. Emerg Med J. Aug 2013;30(8):638-645. [doi: ] [Medline: 22906702]

15. Erenler AK, Akbulut S, Guzel M, et al. Reasons for overcrowding in the emergency department: experiences and suggestions of an education and research hospital. Turk J Emerg Med. Jun 2014;14(2):59-63. [doi: ] [Medline: 27331171]

16. Weiss SJ, Rogers DB, Maas F, Ernst AA, Nick TG. Evaluating community ED crowding: the Community ED overcrowding scale study. Am J Emerg Med. Nov 2014;32(11):1357-1363. [doi: ] [Medline: 25234796]

17. Patel PB, Combs MA, Vinson DR. Reduction of admit wait times: the effect of a leadership-based program. Acad Emerg Med. Mar 2014;21(3):266-273. [doi: ] [Medline: 24628751]

18. Di Somma S, Paladino L, Vaughan L, Lalle I, Magrini L, Magnanti M. Overcrowding in emergency department: an international issue. Intern Emerg Med. Mar 2015;10(2):171-175. [doi: ] [Medline: 25446540]

19. Aksel G, Bildik F, Demircan A, et al. Effects of fast-track in a university emergency department through the National Emergency Department Overcrowding Study. J Pak Med Assoc. Jul 2014;64(7):791-797. [Medline: 25255588]

20. Doan Q, Genuis ED, Yu A. Trends in use in a Canadian pediatric emergency department. CJEM. Sep 2014;16(5):405-410. [doi: ] [Medline: 25227649]

21. Korsten P, Sliwa B, Kühn M, Müller GA, Blaschke S. Impact of professional quality management on interdisciplinary emergency care units. Eur J Emerg Med. Apr 2014;21(2):98-104. [doi: ] [Medline: 24573191]

22. Handel DA, Fu R, Vu E, et al. Association of emergency department and hospital characteristics with elopements and length of stay. J Emerg Med. Jun 2014;46(6):839-846. [doi: ] [Medline: 24462026]

23. Sullivan CM, Staib A, Flores J, et al. Aiming to be NEAT: safely improving and sustaining access to emergency care in a tertiary referral hospital. Aust Health Rev. Nov 2014;38(5):564-574. [doi: ] [Medline: 25297518]

24. Wang H, Robinson RD, Bunch K, et al. The inaccuracy of determining overcrowding status by using the national ED overcrowding study tool. Am J Emerg Med. Oct 2014;32(10):1230-1236. [doi: ] [Medline: 25176566]

25. Jo S, Jin YH, Lee JB, Jeong T, Yoon J, Park B. Emergency department occupancy ratio is associated with increased early mortality. J Emerg Med. Feb 2014;46(2):241-249. [doi: ] [Medline: 23992849]

26. Khanna S, Boyle J, Zeitz K. Using capacity alert calls to reduce overcrowding in a major public hospital. Aust Health Rev. Jun 2014;38(3):318-324. [doi: ] [Medline: 24814040]

27. Handel DA, French LK, Nichol J, Momberger J, Fu R. Associations between patient and emergency department operational characteristics and patient satisfaction scores in an adult population. Ann Emerg Med. Dec 2014;64(6):604-608. [doi: ] [Medline: 25182541]

28. Xu Y, Ho V. Freestanding emergency departments in Texas do not alleviate congestion in hospital-based emergency departments. Am J Emerg Med. Mar 2020;38(3):471-476. [doi: ] [Medline: 31126669]

29. Strada A, Bravi F, Valpiani G, Bentivegna R, Carradori T. Do health care professionals’ perceptions help to measure the degree of

overcrowding in the emergency department? A pilot study in an Italian University hospital. BMC Emerg Med. Aug 27, 2019;19(1):47. [doi: ] [Medline: 31455226]

30. Ajmi F, Zgaya H, Othman SB, Hammadi S. Agent-based dynamic optimization for managing the workflow of the patient’s pathway. Simul Model Pract Theory. Nov 2019;96:101935. [doi: ]

31. Forero R, Man N, Ngo H, et al. Impact of the four-hour National Emergency Access Target on 30 day mortality, access block and chronic emergency department overcrowding in Australian emergency departments. Emerg Medicine Australasia. Feb 2019;31(1):58-66. [doi: ] [Medline: 30062847]

32. Doan Q, Wong H, Meckler G, et al. The impact of pediatric emergency department crowding on patient and health care system outcomes: a multicentre cohort study. CMAJ. Jun 10, 2019;191(23):E627-E635. [doi: ] [Medline: 31182457]

33. van der Linden MCC, van Ufford H, Project Group Medical Specialists, van der Linden NN. The impact of a multimodal intervention on emergency department crowding and patient flow. Int J Emerg Med. Aug 27, 2019;12(1):21. [doi: ] [Medline: 31455260]

34. Spencer S, Stephens K, Swanson-Biearman B, Whiteman K. Health care provider in triage to improve outcomes. J Emerg Nurs. Sep 2019;45(5):561-566. [doi: ] [Medline: 30827577]

35. Berg LM, Ehrenberg A, Florin J, Östergren J, Discacciati A, Göransson KE. Associations between crowding and ten-day mortality among patients allocated lower triage acuity levels without need of acute hospital care on departure from the emergency department. Ann Emerg Med. Sep 2019;74(3):345-356. [doi: ] [Medline: 31229391]

36. Boyle LM, Marshall AH, Mackay M. Coxian phase-type regression models for understanding the relationship between patient attributes, overcrowding, and length of stay in hospital emergency departments. Presented at: 4th International Conference on Health Care Systems Engineering, HCSE 2019. May 30 to Jun 1, 2019:Springer. 53-64; Montréal, Canada. 2020.[doi: ]

37. Torto AD, Pozzi R, Porazzi E, Garagiola E, Strozzi F. Length of stay reduction in the emergency department and its quantification using

complex network theory. IJOR. 2019;36(3):337. [doi: ]

38. Berg LM, Ehrenberg A, Florin J, Östergren J, Göransson KE. Significant changes in emergency department length of stay and case mix over eight years at a large Swedish University Hospital. Int Emerg Nurs. Mar 2019;43:50-55. [doi: ] [Medline: 30190224]

39. Acuna JA, Zayas-Castro JL, Charkhgard H. Ambulance allocation optimization model for the overcrowding problem in US emergency departments: a case study in Florida. Socioecon Plann Sci. Sep 2020;71:100747. [doi: ]

40. Davis Z, Zobel CW, Khansa L, Glick RE. Emergency department resilience to disaster-level overcrowding: a component resilience framework for analysis and predictive modeling. J of Ops Management. Jan 2020;66(1-2):54-66. [doi: ]

41. Menon NVB, Jayashree M, Nallasamy K, Angurana SK, Bansal A. Bed utilization and overcrowding in a high-volume tertiary level pediatric emergency department. Indian Pediatr. Aug 15, 2021;58(8):723-725. [doi: ] [Medline: 33634795]

42. Cirillo W, Freitas LRC, Kitaka EL, et al. Impact of emergency short-stay unit opening on in-hospital global and cardiology indicators. Evaluation Clinical Practice. Dec 2021;27(6):1262-1270. [doi: ] [Medline: 33421284]

43. Savioli G, Ceresa IF, Novelli V, Ricevuti G, Bressan MA, Oddone E. How the coronavirus disease 2019 pandemic changed the patterns of healthcare utilization by geriatric patients and the crowding: a call to action for effective solutions to the access block. Intern Emerg Med. Mar 2022;17(2):503-514. [doi: ] [Medline: 34106397]

44. Gorski JK, Arnold TS, Usiak H, Showalter CD. Crowding is the strongest predictor of left without being seen risk in a pediatric emergency department. Am J Emerg Med. Oct 2021;48:73-78. [doi: ] [Medline: 33845424]

45. Kegel F, Luo OD, Richer S. The impact of extreme heat events on emergency departments in Canadian hospitals. Wilderness Environ Med. Dec 2021;32(4):433-440. [doi: ] [Medline: 34364750]

46. Al-Qahtani MF, Khubrani FY. Exploring potential association between emergency department crowding status and patients’ length of stay

at a university hospital in Saudi Arabia. Open Access Emerg Med. 2021;13:257-263. [doi: ] [Medline: 34188561]

47. Kenny E, Hassanzadeh H, Khanna S, Boyle J, Louise S. Patient flow simulation using historically informed synthetic data. In: Studies in Health Technology and Informatics. Vol 276. IOS Press; 2021:32-37. [doi: ]

48. Trotzky D, Posner L, Mosery J, Cohen A, Avisar S, Pachys G. Do automatic push notifications improve patient flow in the emergency department? Analysis of an ED in a large medical center in Israel. PLoS ONE. 2021;16(10):e0258169. [doi: ] [Medline: 34618849]

49. Wretborn J, Starkenberg H, Ruge T, Wilhelms DB, Ekelund U. Validation of the modified Skåne emergency department assessment of patient load (mSEAL) model for emergency department crowding and comparison with international models; an observational study. BMC Emerg Med. Feb 22, 2021;21(1):21. [doi: ] [Medline: 33618658]

50. Le DX, Do HT, Bui KT, et al. Lean management for improving hospital waiting times-case study of a Vietnamese public/general hospital emergency department. Int J Health Plann Manage. Jan 2022;37(1):156-170. [doi: ] [Medline: 34490656]

51. Theodoro D, Vyas N, Ablordeppey E, et al. Central venous catheter adverse events are not associated with crowding indicators. W J Emerg Med. 2020;22(2). [doi: ] [Medline: 33856335]

52. Soares VS. Analysis of the internal bed regulation committees from hospitals of a Southern Brazilian city. Einstein (Sao Paulo). 2017;15(3):339-343. [doi: ] [Medline: 29091157]

53. Phillips JL, Jackson BE, Fagan EL, et al. Overcrowding and Its association with patient outcomes in a median-low volume emergency department. J Clin Med Res. Nov 2017;9(11):911-916. [doi: ] [Medline: 29038668]

54. Wang H, Ojha RP, Robinson RD, et al. Optimal measurement interval for emergency department crowding estimation tools. Ann Emerg Med. Nov 2017;70(5):632-639. [doi: ] [Medline: 28688771]

55. Burke JA, Greenslade J, Chabrowska J, et al. Two hour evaluation and referral model for shorter turnaround times in the emergency

department. Emerg Med Australas. Jun 2017;29(3):315-323. [doi: ] [Medline: 28455884]

56. Street M, Mohebbi M, Berry D, Cross A, Considine J. Influences on emergency department length of stay for older people. Eur J Emerg Med. Aug 2018;25(4):242-249. [doi: ] [Medline: 28151752]

57. Truong M, Meckler G, Doan QH. Emergency department return visits within a large geographic area. J Emerg Med. Jun 2017;52(6):801-808. [doi: ] [Medline: 28228344]

58. Georgio G, Guttmann A, Doan QH. Emergency department flow measures for adult and pediatric patients in British Columbia and Ontario: a retrospective, repeated cross-sectional study. J Emerg Med. Sep 2017;53(3):418-426. [doi: ] [Medline: 28676415]

59. Landa P, Sonnessa M, Resta M, Tànfani E, Testi A. A hybrid simulation approach to analyse patient boarding in emergency departments. In: Health Care Systems Engineering. Springer; 2017:133-144. [doi: ]

60. Chiu IM, Lin YR, Syue YJ, Kung CT, Wu KH, Li CJ. The influence of crowding on clinical practice in the emergency department. Am J Emerg Med. Jan 2018;36(1):56-60. [doi: ] [Medline: 28705743]

61. Rahmani F, Rezazadeh F, Ala A, Soleimanpour M, Mehdizadeh Esfanjani R, Soleimanpour H. Evaluation of overcrowding of emergency department in Imam Reza Hospital in 2015 by implementing 2 scales: NEDOCS and EDWIN. Iran Red Crescent Med J. 2017;19(6):1-4. [doi: ]

62. Scott I, Sullivan C, Staib A, Bell A. Deconstructing the 4-h rule for access to emergency care and putting patients first. Aust Health Rev. Dec 2018;42(6):698-702. [doi: ] [Medline: 29032791]

63. Dai JG, Shi P. A two-time-scale approach to time-varying queues in hospital inpatient flow management. Oper Res. Apr 2017;65(2):514-536. [doi: ]

64. Santos E, Cardoso D, Queirós P, Cunha M, Rodrigues M, Apóstolo J. The effects of emergency department overcrowding on admitted patient outcomes: a systematic review protocol. JBI Database System Rev Implement Rep. May 2016;14(5):96-102. [doi: ] [Medline: 27532467]

65. Ming T, Lai A, Lau PM. Can team triage improve patient flow in the emergency department? A systematic review and meta-analysis. Adv Emerg Nurs J. 2016;38(3):233-250. [doi: ] [Medline: 27482995]

66. Burström L, Engström ML, Castrén M, Wiklund T, Enlund M. Improved quality and efficiency after the introduction of physician-led team triage in an emergency department. Ups J Med Sci. 2016;121(1):38-44. [doi: ] [Medline: 26553523]

67. Bucci S, de Belvis AG, Marventano S, et al. Emergency department crowding and hospital bed shortage: is lean a smart answer? A systematic review. Eur Rev Med Pharmacol Sci. Oct 2016;20(20):4209-4219. [Medline: 27831655]

68. Fuentes E, Shields JF, Chirumamilla N, et al. “One-way-street” streamlined admission of critically ill trauma patients reduces emergency department length of stay. Intern Emerg Med. Oct 2017;12(7):1019-1024. [doi: ] [Medline: 27473424]

69. Li CJ, Syue YJ, Lin YR, et al. Influence of CT utilisation on patient flow in the emergency department: a retrospective 1-year cohort study. BMJ Open. May 4, 2016;6(5):e010815. [doi: ] [Medline: 27147387]

70. Boyle A, Abel G, Raut P, et al. Comparison of the International Crowding Measure in Emergency Departments (ICMED) and the National Emergency Department Overcrowding Score (NEDOCS) to measure emergency department crowding: pilot study. Emerg Med J. May 2016;33(5):307-312. [doi: ] [Medline: 26739294]

71. Ghanes K, Jouini O, Wargon M, Jemai Z. Modeling and analysis of triage nurse ordering in emergency departments. Presented at: 2015 International Conference on Industrial Engineering and Systems Management (IESM); Oct 21, 2015:228-235; Seville, Spain. [doi: ]

72. Cocke S, Guinn D, MacBlane E, et al. UVA emergency department patient flow simulation and analysis. Presented at: 2016 Systems and Information Engineering Design Symposium (SIEDS). Apr 29, 2016:IEEE. 118-123; Charlottesville, VA, USA. 2016.[doi: ]

73. Ngo H, Forero R, Mountain D, et al. Impact of the four-hour rule in Western Australian hospitals: trend analysis of a large record linkage study 2002-2013. PLoS ONE. 2018;13(3):e0193902. [doi: ] [Medline: 29538401]

74. Li M, Vanberkel P, Carter AJE. A review on ambulance offload delay literature. Health Care Manag Sci. Dec 2019;22(4):658-675. [doi: ] [Medline: 29982911]

75. Thapa RR, Bhuiyan M, Krishna A, Prasad PWC. Application of RFID technology to reduce overcrowding in hospital emergency departments. In: Advances in Information Systems Development: Methods, Tools and Management. Springer; 2018:17-32. [doi: ]

76. Andrews R, Suriadi S, Wynn M, Rothwell S. Improving patient flows at St. Andrew's War Memorial Hospital's emergency department through process mining. In: Business Process Management Cases: Digital Innovation and Business Transformation in Practice. Vol Part F612. Springer; 2018:311-333. [doi: ]

77. Wallingford G, Joshi N, Callagy P, Stone J, Brown I, Shen S. Introduction of a horizontal and vertical split flow model of emergency department patients as a response to overcrowding. J Emerg Nurs. Jul 2018;44(4):345-352. [doi: ] [Medline: 29169818]

78. Salehi L, Phalpher P, Valani R, et al. Emergency department boarding: a descriptive analysis and measurement of impact on outcomes. CJEM. Nov 2018;20(6):929-937. [doi: ] [Medline: 29619913]

79. van der Veen D, Remeijer C, Fogteloo AJ, Heringhaus C, de Groot B. Independent determinants of prolonged emergency department length of stay in a tertiary care centre: a prospective cohort study. Scand J Trauma Resusc Emerg Med. Sep 20, 2018;26(1):81. [doi: ] [Medline: 30236125]

80. Molla M, Warren DS, Stewart SL, Stocking J, Johl H, Sinigayan V. A lean six sigma quality improvement project improves timeliness of discharge from the hospital. Jt Comm J Qual Patient Saf. Jul 2018;44(7):401-412. [doi: ] [Medline: 30008352]

81. Kim DU, Park YS, Park JM, et al. Influence of overcrowding in the emergency department on return visit within 72 hours. J Clin Med. May 9, 2020;9(5):1406. [doi: ] [Medline: 32397560]

82. d'Etienne JP, Zhou Y, Kan C, et al. Two-step predictive model for early detection of emergency department patients with prolonged stay and its management implications. Am J Emerg Med. Feb 2021;40:148-158. [doi: ] [Medline: 32063427]

83. Shah R, Leno R, Sinert R. Impact of provider-in-triage in a safety-net hospital. J Emerg Med. Sep 2020;59(3):459-465. [doi: ] [Medline: 32595053]

84. Wachtel G, Elalouf A. Addressing overcrowding in an emergency department: an approach for identifying and treating influential factors and a real-life application. Isr J Health Policy Res. Sep 2, 2020;9(1):37. [doi: ] [Medline: 32873328]

85. Peng Q, Yang J, Strome T, Weldon E, Chochinov A. Evaluation of physician in triage impact on overcrowding in emergency department using discrete-event simulation. 105267/jjpm. 2020;5(4):211-226. [doi: ]

86. Choi Y, Jeong J, Kim BG. Admission Decisions Made by Emergency Physicians Can Reduce the Emergency Department Length of Stay for Medical Patients. Emerg Med Int. 2020;2020(1):8392832. [doi: ] [Medline: 32104606]

87. Payne AS, Brown KM, Berkowitz D, et al. Improving throughput for mid-acuity patients in the pediatric emergency department. Pediatric Quality & Safety. 2020;5(3):e302. [doi: ]

88. Tsai JCH, Weng SJ, Liu SC, et al. Adjusting daily inpatient bed allocation to smooth emergency department occupancy variation. Healthcare (Basel). Mar 28, 2020;8(2):78. [doi: ] [Medline: 32231146]

89. Hoot NR, Banuelos RC, Chathampally Y, Robinson DJ, Voronin BW, Chambers KA. Does crowding influence emergency department treatment time and disposition? J Am Coll Emerg Physicians Open. Feb 2021;2(1):e12324. [doi: ] [Medline: 33521777]

90. Khubrani FY, Al-Qahtani MF. Association between emergency department overcrowding and mortality at a teaching hospital in Saudi Arabia. TOPHJ. Dec 31, 2020;13(1):756-762. [doi: ]

91. Bélanger V, Lahrichi N, Lanzarone E, Yalçındağ S. Health Care Systems Engineering. Springer; 2020. [doi: ]

92. Marsilio M, Roldan ET, Salmasi L, Villa S. Operations management solutions to improve ED patient flows: evidence from the Italian NHS. BMC Health Serv Res. Jul 30, 2022;22(1):974. [doi: ] [Medline: 35908053]

93. Otto R, Blaschke S, Schirrmeister W, Drynda S, Walcher F, Greiner F. Length of stay as quality indicator in emergency departments: analysis of determinants in the German Emergency Department Data Registry (AKTIN registry). Intern Emerg Med. Jun 2022;17(4):1199-1209. [doi: ] [Medline: 34989969]

94. Tseng WM, Lin PH, Wu PC, Kao CH. Examining patient flow in a tertiary hospital’s emergency department at a low coronavirus prevalence region. BMC Emerg Med. Jul 27, 2022;22(1):137. [doi: ] [Medline: 35896981]

95. Kadri F, Dairi A, Harrou F, Sun Y. Towards accurate prediction of patient length of stay at emergency department: a GAN-driven deep learning framework. J Ambient Intell Humaniz Comput. Feb 3, 2022;14(9):1-15. [doi: ] [Medline: 35132336]

96. Badr S, Nyce A, Awan T, Cortes D, Mowdawalla C, Rachoin JS. Measures of emergency department crowding, a systematic review. How to make sense of a long list. Open Access Emerg Med. 2022;14:5-14. [doi: ] [Medline: 35018125]

97. Noris NJ, Putera K, Libasin Z, Krishnan M. Lean healthcare implementation in Malaysian specialist hospitals: challenges and performance evaluation. J Heal Transl Med. 2022;25(1):27-39. [doi: ]

98. Mallows JL. Effects of staff grade, overcrowding and presentations on emergency department performance: a regression model. Emerg Medicine Australasia. Jun 2022;34(3):341-346. [doi: ]

99. Improta G, Majolo M, Raiola E, Russo G, Longo G, Triassi M. A case study to investigate the impact of overcrowding indices in emergency departments. BMC Emerg Med. Aug 9, 2022;22(1):143. [doi: ] [Medline: 35945503]

100. Wretborn J, Ekelund U, B. Wilhelms D. Differentiating properties of occupancy rate and workload to estimate crowding: A Swedish national cross-sectional study. JACEP Open. Feb 2022;3(1):e12648. [doi: ]

101. van der Linden C, Reijnen R, Derlet RW, et al. Emergency department crowding in The Netherlands: managers’ experiences. Int J Emerg Med. Oct 24, 2013;6(1):1-8. [doi: ] [Medline: 24156298]

102. Johnson KD, Winkelman C. The effect of emergency department crowding on patient outcomes: a literature review. Adv Emerg Nurs J. 2011;33(1):39-54. [doi: ] [Medline: 21317697]

103. de Araujo P, Khraiche M, Tukan A. Does overcrowding and health insurance type impact patient outcomes in emergency departments? Health Econ Rev. Nov 12, 2013;3(1):1-7. [doi: ] [Medline: 24229451]

104. Wang H, Robinson RD, Garrett JS, et al. Use of the SONET score to evaluate high volume emergency department overcrowding: a prospective derivation and validation study. Emerg Med Int. 2015;2015:401757. [doi: ] [Medline: 26167302]

105. Lee YJ, Shin SD, Lee EJ, Cho JS, Cha WC. Emergency department overcrowding and ambulance turnaround time. PLoS ONE. 2015;10(6):e0130758. [doi: ] [Medline: 26115183]

106. Lin CH, Kao CY, Huang CY. Managing emergency department overcrowding via ambulance diversion: a discrete event simulation model. J Formos Med Assoc. Jan 2015;114(1):64-71. [doi: ] [Medline: 25618586]

107. Konrad R, DeSotto K, Grocela A, et al. Modeling the impact of changing patient flow processes in an emergency department: insights from a computer simulation study. Oper Res Health Care. Dec 2013;2(4):66-74. [doi: ]

108. Elalouf A, Wachtel G. An alternative scheduling approach for improving patient-flow in emergency departments. Oper Res Health Care. Dec 2015;7:94-102. [doi: ]

109. Kim BBJ, Delbridge TR, Kendrick DB. Improving process quality for pediatric emergency department. Int J Health Care Qual Assur. 2014;27(4):336-346. [doi: ] [Medline: 25076607]

110. Fayyaz J, Khursheed M, Mir MU, Mehmood A. Missing the boat: odds for the patients who leave ED without being seen. BMC Emerg Med. Jan 16, 2013;13(1):1-9. [doi: ] [Medline: 23324162]

111. Vegting IL, Alam N, Ghanes K, et al. What are we waiting for? Factors influencing completion times in an academic and peripheral

emergency department. Neth J Med. Aug 2015;73(7):331-340. [Medline: 26314716]

112. Tekwani KL, Kerem Y, Mistry CD, Sayger BM, Kulstad EB. Emergency department crowding is associated with reduced satisfaction scores in patients discharged from the emergency department. West J Emerg Med. Feb 2013;14(1):11-15. [doi: ] [Medline: 23447751]

113. Kang J, Kim J, Jo YH, et al. ED crowding and the outcomes of out-of-hospital cardiac arrest. Am J Emerg Med. Nov 2015;33(11):1659-1664. [doi: ] [Medline: 26324002]

114. Savioli G, Ceresa IF, Bressan MA, et al. Five level triage vs. four level triage in a quaternary emergency department: national analysis on waiting time, validity, and crowding-the CREONTE (Crowding and RE-Organization National TriagE) study group. Medicina (Kaunas). Apr 17, 2023;59(4):781. [doi: ] [Medline: 37109739]

115. Watson A, Stuart WP. Improving safety and quality with an emergency department overcrowding plan. J Emerg Nurs. Sep 2023;49(5):680-693. [doi: ] [Medline: 37409998]

116. Hsieh A, Arena A, Oraha A, et al. Implementation of vertical split flow model for patient throughput at a community hospital emergency department. J Emerg Med. Jan 2023;64(1):77-82. [doi: ] [Medline: 36641257]

117. Van Der Linden MC, Van Loon-Van Gaalen M, Richards JR, Van Woerden G, Van Der Linden N. Effects of process changes on emergency department crowding in a changing world: an interrupted time-series analysis. Int J Emerg Med. Feb 15, 2023;16(1):6. [doi: ] [Medline: 36792991]

118. Hsu NC, Shu CC, Lin YF, Yang MC, Su S, Ko WJ. Why do general medical patients have a lengthy wait in the emergency department before admission? J Formos Med Assoc. Aug 2014;113(8):557-561. [doi: ] [Medline: 25037761]

119. Hofer KD, Saurenmann RK. Parameters affecting length of stay in a pediatric emergency department: a retrospective observational study. Eur J Pediatr. May 2017;176(5):591-598. [doi: ] [Medline: 28275860]

120. Schmutz T, Le Terrier C, Ribordy V, Guechi Y. No waiting lying in a corridor: a quality improvement initiative in an emergency department. BMJ Open Qual. Aug 2023;12(3):e002431. [doi: ] [Medline: 37640478]

121. George G, Jell C, Todd BS. Effect of population ageing on emergency department speed and efficiency: a historical perspective from a district general hospital in the UK. Emerg Med J. May 2006;23(5):379-383. [doi: ] [Medline: 16627841]
